# Systematic review on effects of experimental orthodontic tooth displacement on brain activation assessed by fMRI

**DOI:** 10.1002/cre2.879

**Published:** 2024-04-01

**Authors:** Gelareh Sadvandi, Amir Ehsan Kianfar, Kathrin Becker, Alexander Heinzel, Michael Wolf, Sareh Said‐Yekta Michael

**Affiliations:** ^1^ Department of Orthodontics RWTH Aachen University Hospital Germany; ^2^ RWTH Aachen University Germany; ^3^ Department of Dentofacial Orthopedics and Orthodontics Charité Universitätsmedizin Berlin Berlin CC03 Germany; ^4^ Department of Nuclear Medicine Martin‐Luther‐University Halle‐Wittenberg Halle Germany

**Keywords:** magnetic resonance imaging, brain activity, orthodontic treatment, orthodontic tooth movement

## Abstract

**Background:**

Orthodontic treatment is often accompanied by discomfort and pain in patients, which are believed to be a result of orthodontic tooth displacement caused by the mechanical forces exerted by the orthodontic appliances on the periodontal tissues. These lead to change blood oxygen level dependent response in related brain regions.

**Objective:**

This systematic review aims to assess the impact of experimental orthodontic tooth displacement on alterations in central nervous system activation assessed by tasked based and resting state fMRI.

**Materials and Methods:**

A literature search was conducted using online databases, following PRISMA guidelines and the PICO framework. Selected studies utilized magnetic resonance imaging to examine the brain activity changes in healthy participants after the insertion of orthodontic appliances.

**Results:**

The initial database screening resulted in 791 studies. Of these, 234 were duplicates and 547 were deemed irrelevant considering the inclusion and exclusion criteria. Of the ten remaining potential relevant studies, two were excluded during full‐text screening. Eight prospective articles were eligible for further analysis. The included studies provided evidence of the intricate interplay between orthodontic treatment, pain perception, and brain function. All of the participants in the included studies employed orthodontic separators in short‐term experiments to induce tooth displacement during the early stage of orthodontic treatment. Alterations in brain activation were observed in brain regions, functional connectivity and brain networks, predominantly affecting regions implicated in nociception (thalamus, insula), emotion (insula, frontal areas), and cognition (frontal areas, cerebellum, default mode network).

**Conclusions:**

The results suggest that orthodontic treatment influences beyond the pain matrix and affects other brain regions including the limbic system. Furthermore, understanding the orthodontically induced brain activation can aid in development of targeted pain management strategies that do not adversely affect orthodontic tooth movement. Due to the moderate to serious risk of bias and the heterogeneity among the included studies, further clinical trials on this subject are recommended.

## 1 INTRODUCTION

Orthodontic tooth movement (OTM) has been categorized into four distinct phases based on the rate of tooth displacement: the initial phase, lag phase, acceleration phase, and linear phase. The initial phase is triggered immediately upon the application of mechanical forces and persists for a duration of 24 h to 48 h (Asiry, 2018; Behm et al., 2022). This phase is characterized by immediate tooth displacement within the periodontal space and is associated with patient‐reported pain and discomfort (Bergius et al., 2000; Zhang et al., 2021). These adverse experiences are a leading factor for the termination of orthodontic treatment. Therefore, further scientific investigation in this area is imperative to prevent the treatment discontinuation. Tooth movement can be induced physiologically or orthodontically by applying vertical or horizontal stimulation to the periodontal ligament (PDL), the connective tissue surrounding the tooth root, which adjacent alveolar bone, triggering a biological response that culminates in the remodeling of these tissues (Isola et al., 2016; Wichelhaus & Eichenberg, 2017). The application of orthodontic force on PDL leads to vascular changes and ischemia, as well as displacement and deformation of the tissue. These changes lead to inflammatory reactions in the periodontium, which stimulate the release of various biochemical mediators. The nociceptive information is transmitted to the cerebral cortex, where it is subjectively perceived as pain (Fleming et al., 2016; Krishnan, 2007) and manifests as activity changes in the corresponding brain areas (Lavigne & Sessle, 2016).

Nociceptive pathways play an important role in conveying pain signals emanating from dental and periodontal tissues during orthodontic treatment. The afferent signals, initiated by sensory receptors in the dental pulp and PDL due to orthodontic apparatus, traverse through the trigeminal ganglion, spinal trigeminal nucleus, and thalamus. The anterior cingulate cortex (ACC) receives inputs from both the medial thalamus and primary somatosensory cortex, integrating the nociceptive information and regulating the aversive response to pain. These signals ultimately reach the somatosensory cortex where pain is perceived. Subsequent efferent signals then regulate bone metabolism in periodontal structures, facilitated by the sympathetic nervous system. The descending nociceptive pathway, with origins in the cortex, modulates these signals, influencing spinal cord neurons to either amplify or attenuate pain transmission (Ariji et al., 2019).

Orofacial pain and discomfort can be investigated by studying brain activity changes (Lin, 2014). Magnetic Resonance Imaging (MRI) is a safe and effective method that can measure the blood Oxygen Level‐Dependent (BOLD) contrast to observe objective brain activities (Lin, 2014; Miranda et al., 2021). The BOLD signal is generated by changes in the ratio of oxyhemoglobin to deoxyhemoglobin, which is caused by temporal hypoxia around the neurons during their activation, leading to increased blood flow (Stonier & Hardee, 2018). The Amplitude of Low‐Frequency Fluctuation (ALFF) and fractional ALFF (fALFF) are both methods used to evaluate the resting state functional Magnetic Resonance Imaging (rs‐fMRI) data. ALFF measures the intensity of spontaneous brain activity by comparing the scale of the raw signal to the arbitrary signal BOLD. At the same time, fALFF provides a standardized solution by considering the ratio of ALFF to the total amplitude within the entire frequency band. Functional connectivity (FC) analysis is also commonly utilized to investigate spontaneous neuronal activity's functional integration by calculating temporal correlation. These methods can provide complementary information about resting‐state brain activity and help in the understanding of the functional organization and dynamics of the brain (Jia et al., 2020; Zou et al., 2008).

Orthodontic appliances can be classified into fixed or removable types. Fixed appliances are more commonly used, causing more pain compared to removable ones (Krishnan, 2007). It is worth noting that both types can be fabricated using either metallic or non‐metallic components. However, metal or ferromagnetic materials can affect the magnetic fields of neuroimaging devices, leading to image artifacts (Kajan et al., 2015). They can also be dangerous when exposed to magnetic fields, causing thermal issues. Furthermore, the ferromagnetic components can be absorbed by the magnetic fields (Stonier & Hardee, 2018). Therefore, elastomeric separators have emerged as the prevailing orthodontic appliance in MRI studies (Abu Al‐Melh & Andersson, 2017; Zhang et al., 2021) in many clinical trials, (Marini et al., 2013; Michelotti et al., 1999) to mitigate these risks. Separators are primarily employed to generate space between adjacent teeth, facilitating the precise positioning of orthodontic bands.

The current systematic review (SR) aims to provide an overview of structural and functional brain neuroimaging studies that sought alteration in brain activities in the first stage of OTM.

## 2 MATERIALS AND METHODS

### 2.1 Protocol development

This SR follows the guidelines of the Preferred Reporting Items for systematic reviews and Meta‐Analyses (PRISMA) (Appendix 1: PRISMA_2020_checklist) (Marini et al., 2013), and the review protocol was registered in the international prospective register of systematic reviews (PROSPERO) database with the ID number CRD42022303910.

### 2.2 Search strategy and study selection

In this SR, two reviewers (GS and AK) carried out an extensive search on four electronic databases PubMed, EMBASE, Web of Science, and Cochrane Central Register of Controlled Trials (CENTRAL) via the Cochrane Library. The reviewers performed the search independently and updated the electronic databases on September 28th, 2023.

The reviewers (GS and AK) assessed the eligibility of the studies independently for inclusion by conducting an initial screening based on the titles and abstracts, followed by a full‐text screening. Discussions with other reviewers (SM and KB) resolved disagreements in study selection. If clarification or additional data were required, the authors of the respective studies were contacted via email.

The reviewers searched the articles with a combination of the keywords “brain”, “cerebrum”, “central nervous system”, “CNS”, orthodont*, “orthodontic force”, “tooth movement*”, “tooth mobility”, “dental orthopedic*”, “tooth retraction”, “tooth migration”, “tooth displacement”, “magnetic resonance imaging”, “MRI”, “fMRI” using the Boolean operators “AND” and “OR” and using the MeSH terms and free text in all fields in the databases mentioned above, independently (Appendix 2: Database_Search_Strategy).

Relevant publications were selected based on predetermined inclusion and exclusion criteria. Inclusion criteria consisted clinical trials conducted on healthy individuals with no restrictions regarding language. Exclusion criteria consisted of animal studies, review articles, studies involving participants with chronic orofacial pain, Central Nervous System (CNS) disorders, bone metabolism disorders, medical orofacial illness, temporomandibular disorders or temporomandibular joint disorders, severe acute or chronic pain, chronic medical conditions, psychiatric disorders, and autoimmune disorders. The search strategy, inclusion, exclusion criteria and the main research question were applied to identify eligible studies, which were defined in PICO format (The Guidelines Manual, 2012) as follows:

Population: Healthy individuals without previous orthodontic treatment.

Intervention: Subject to experimental orthodontic tooth displacement.

Comparison: Post‐intervention versus pre‐intervention changes in the same individuals or comparison of individuals in the intervention group with those in the control group.

Outcome: Activation patterns of the brain.

### 2.3 Data collection and extraction

The searched studies from the databases were imported into Covidence software (Covidence SR software, Veritas Health Innovation, Melbourne, Australia) for data extraction, and removing the duplicates by two reviewers (AK and GS), independently. The inclusion and exclusion criteria were applied within the software, and the reference lists of the included studies were scrutinized. The data summary tables were filled with information relevant to the PICO characteristics, including the last name of the first author, publication year, study design, the country in which the study was conducted, participant demographics (number, gender, and age range), comparison characteristics, and covariates in Table 1 and additional data collected is presented in Table 2, including the type of intervention, treated teeth, duration of intervention, task, imaging modality, analysis methods, motion correction methods, and neuroimaging findings.

**Table 1:** Demography and overview of the included studies.

ID #	Study			Intervention group characteristics		Comparison characteristics	Covariates
	Author, publication year	Design	Location	Sample size: total, final (female‐male)	Age, mean ± SD, handedness	Type, age, gender, sample size, handedness	
1	Ariji et al. (2018)	prospective without control group	Japan	10, 10: (4f – 6 m)	26−40 year mean: 30.5 ± 5.9 N/A	baseline (at rest: 60 s before separator insertion) handedness N/A	Not reported
2	Ariji et al. (2019)	prospective without control group	Japan	10, 10: (4 f−6 m)	mean: 30.8 N/A	1. baseline: (60 s before insertion) in the separator group 2. rest (60 s during no biting) in the separator group with biting 3. low‐level clenching group 6 (2 f−4 m), mean age: 38.5 handedness N/A	Not reported
3	Jin et al. (2021)	prospective with control group	China	49, 44: (24 f–20 m)	mean: 21.0 ± 0.9 right‐handed	control group (age‐sex‐matched): without separator 49 (27 f–22 m) mean age: 21.0 ± 2.6 right‐handed	Age/sex/frame‐wise displacement
4	Maurer et al. (2021) Kondo et al. (2013)	prospective without control group	Germany	19, 19: (0 f–19 m)	mean: 25.7 ± 2.8 right‐handed	1. at rest 20−30 s 2. same participant (with clenching) before separator placement	STAI‐state
5	Yang et al. (2015)	prospective without control group	China	17, 15: (15 f–0 m)	18−24 year mean: 21.4 right‐handed	same participant before insertion of separators	head motions/global mean signal/WM and CSF signals
6	Zhang et al. (2020)	prospective with control group	China	48, 44: (24 f–20 m)	19–23 year mean: 21.0 ± 0.9 right‐handed	control group: (age‐sex‐matched): without separator 49 (27 f–22 m) age:19−30 mean age: 21 ± 2.6 right‐handed	age/sex
7	Jin et al. (2022)	prospective with control group	China	49,44: (24– 20)	age:18‐45 year mean age: 21.0 ± 0.9 right‐handed	control group: (age‐sex‐matched): without separator 49 (27−22) age:18‐45 year mean age: 21.6 ± 0.9 right‐handed	Age/sex/frame‐wise displacement
8	Zhang et al. (2022)	prospective with control group	China	52,48 (27f‐21 m)	age:18‐24 years mean age: 21.0 ± 1.1 right‐handed	control group: (age‐sex‐matched): 49 (27 f,22 m) age:19−30 years mean age: 21.6 ± 2.6 right‐handed	age/sex

**Table 2:** Experimental design and major neuroimaging findings of the included studies.

ID #	Study (author, publication year)	Type of Orthodontic device/size	Type of treated teeth	Duration of intervention/Task	Image modality/Analysis method	Motion Correction Methods	Main Neuroimaging Findings: Significant Alteration in Brain Functional Activation/FC	RoB Results
1	Ariji et al. (2018)	‐ alt. elastomeric separator: floss with wax ‐ alt. brass wire separator: brass contact gauge 0.15‐ or 0.20‐mm diameter	maxillary right first and second premolars	30 s (in MRI)/‐	fMRI: BOLD whole‐brain	‐Head fixed ‐Functional images realigned to remove motion artifacts	‐ **floss compared to baseline: BOLD increase**: in L parietal association area, frontal association, temporal association, insula, cerebellum, hippocampus, amygdala (paired *t*‐test) **‐ brass contact gauge insertion compared to baseline: BOLD increase**: in L parietal association area, L frontal association, L temporal association, L insula, L cerebellum, R thalamus, R hippocampus, R calcarine sulcus, L putamen, L lingual gyrus (paired *t*‐test)	Serious
2	Ariji et al. (2019)	‐ alt. brass wire separator: brass contact gage 0.15‐ or 0.2‐ mm thickness	maxillary right premolars	60 s (in MRI)/5 s biting	fMRI: BOLD whole‐brain	‐Head fixed ‐Functional images realigned to remove motion artifacts	**‐ brass contact gauge compared to baseline: BOLD increase**: in the primary sensorimotor cortex, frontal association area, temporal association area, cerebellum (paired *t*‐test) **‐ brass contact gauge with biting compared to rest: BOLD increase**: (paired *t*‐test) in above‐mentioned areas + parietal association area, lingual gyrus, thalamus, hippocampus/amygdala, putamen, and insula **‐ brass contact gauge with and without biting**: (paired *t*‐test) R parietal association area, R hippocampus/amygdala, and the bilateral parahippocampal gyrus **‐ brass contact gauge with biting compared to low‐level clenching**: (paired *t*‐test) hypothalamus in addition to brain regions activated during low‐level clenching	Serious
3	Jin et al. (2021)	elastic separator 4.0 mm diameter	mesial sides of the left mandibular first molar	24 h/‐	rs‐fMRI: fALFF ROI seed‐based‐FC ROI	‐Regressed out nuisance WM, CSF signals ‐Head motion ≥2.5 mm/◦ excluded ‐Temporal scrubbing for spikes	**elastic separator compared with the control group**: (two‐sample *t*‐test) **‐ fALFF increase**: in dorsal Thalamus ‐ **fALFF decrease**: in medial Thalamus **‐ FC decrease**: medial Thalamus with 12 regions: L cerebellum, bilateral anterior cingulate cortex (ACC), right parahippocampal gyrus, bilateral middle frontal gyrus, bilateral superior frontal gyrus, R inferior frontal gyrus, R middle temporal gyrus, R insula, and R thalamus No FC alteration between the dorsal thalamus and any of the brain regions	Moderate
4	Maurer et al. (2021)	elastic separator 2.1 mm	second bicuspid and the first molar on the right side of mandible	24 h/3 s clenching	t‐fMRI: BOLD whole‐brain	‐six motion parameters ‐Realignment used ‐Data spatially smoothed	**painful tooth clenching compared to rest: BOLD increase**: bilateral anterior and posterior insula, bilateral thalamus, bilateral secondary somatosensory cortex (S2), bilateral inferior frontal gyrus (IFG), bilateral putamen, bilateral inferior parietal lobule (IPL), middle cingulate gyrus (MCC), bilateral middle frontal gyrus (MFG), bilateral superior frontal gyrus (SFG), bilateral cerebellum and L primary motor cortex (M1) (one‐sample *t*‐test) **‐ painful tooth clenching compared to clenching: BOLD increase**: bilateral S1, bilateral S2, bilateral M1, SMA, R rolandic operculum, and bilateral insula (anterior and posterior) (paired *t*‐test)	Moderate
5	Yang et al. (2015)	elastic separators not reported	mesial and distal side of right mandibular first molars	24 h/‐	rs‐fMRI: voxelwise ALFF seed‐based‐FC ROI	‐Head fixed ‐six motion parameters ‐ Regressed out nuisance CSF signals ‐Head motion >1.0 mm/° excluded	**elastic separator compared to normal state**: (two‐sample *t*‐test) **‐ ALLF increase**: L insular cortex and R supplementary motor area. **‐ ALFF decrease**: pyramis‐L and uvula‐R in the bilateral cerebellum posterior lobe, bilateral angular gyrus in parietal lobe/precuneus, and superior frontal gyrus. **‐ FC increase**: between pyramis of the L cerebellum posterior lobe and R parietal lobe (ROI), **‐ FC decrease**: between pyramis of the R cerebellum posterior lobe and L insular cortex (ROI), between L middle temporal gyrus and L precuneus (ROI), between L parietal lobe and L posterior cerebellum (ROI), between cuneus occipital lobe and L posterior cerebellum (ROI)	Serious
6	Zhang et al. (2020)	elastic separator not reported	first and the second molar on the right side of the mandible	24 h/‐	rs‐fMRI/BOLD/whole‐brain FC	‐Head motion corrected ‐24‐parameter motion regressed ‐ High Framewise Displacement Removed ‐Head motion > 2.0 mm/° Excluded	**elastic separator compared with the control group**: (two‐sample *t*‐test) **between GM networks**: **‐ FC increase**: GM3‐GM5‐GM7 **‐ FC decrease**: GM2‐GM3‐GM8 **between WM networks**: **‐ FC increase**: between WM12 and: WM1, WM4, WM14 **‐ FC decrease**: WM1‐WM2‐WM11‐WM3‐WM4‐WM5‐WM11/WM5‐WM12‐WM9 **between GM and WM networks**: **‐ FC increase**: between GM5 and WM4, and in GM6‐WM9‐GM7‐WM2 and between WM12 and GM3, GM4, GM5, GM6, GM7, GM8, GM9, GM11, GM12 **‐ FC decreased**: between GM3 and WM5, between GM8 and WM11, in WM1‐GM10‐WM2 **‐ FC in GM‐WM loops**: alteration in GM5‐WM12‐WM4‐GM5/GM3‐WM12‐WM5‐GM3/GM7‐WM12‐WM9‐GM7*	Moderate
7	Jin et al. (2022)	elastic separator 4 mm	the left side of mandible between second bicuspid and the first molar	24 h/‐	rs‐fMRI: fALFF/ whole‐brain	‐Head motion >2.0 mm/excluded, ‐24‐parameter motion correction;	**elastic separator compared with the control group**: (two‐sample *t*‐test) **fALFF increase**: L cerebellum, R PCC, and bilateral inferior temporal gyrus **fALFF decrease**: middle PFC, the L ACC, bilateral angular gyrus, L inferior parietal cortex, middle temporal gyrus, and miscellaneous cerebral regions	Moderate
8	Zhang et al. (2022)	elastic separator not reported	right first and second molar on the mesial and distal	24 h/‐	rs‐fMRI, whole‐brain network	‐First 10 time‐points removed to stabilize initial signals. ‐Slice‐Timing adjusted: Aligns acquired slices. ‐ Head motion ≥2.5 mm/◦ excluded ‐24‐Parameter motion correction. ‐Nuisance signals regression ‐High FWD removed to filter motion spikes.	**elastic separator compared with the control group**: (Independent‐sample *t*‐test) **global topological organization**: ‐ clustering coefficient decrease ‐ local efficiency decrease **nodal topological organization**: **‐ nodal centralities increase**: mainly ipsilateral (right) brain areas: SFG (R, lateral and middle), precentral gyrus R, parahippocampal gyrus (bilateral entorhinal cortex and R posterior cortex), postcentral gyrus R, insula gyrus, basal ganglia (R caudal hippocampus and L ventral caudate), thalamus (R occipital thalamus and bilateral caudal temporal thalamus) **‐ nodal centralities decrease**: mainly contralateral (left) brain areas: STG (L caudal area), middle temporal gyrus (L dorsolateral area), inferior temporal gyrus (L ventrolateral area), postcentral gyrus, L and R cingulate gyrus (L, middle and R), occipital cortex (L, middle)	Moderate

* Lateral visual network (GM1), anterior lobe of cerebellum network (GM2), dorsal attention network (DAN) (GM3), medial occipital network (GM4), default mode network (DMN) (GM5), superior frontal network (GM6), salience network (SN) (GM7), executive control network (ECN) (GM8), somatomotor network (GM9), posterior lobe of the cerebellum and subcortical network (GM10), orbitofrontal–temporal network (GM11), and middle temporal network (GM12), posterior cingulum (retrosplenial) bundle and angular WM network (WM1), inferior frontal WM network (WM2), corona radiata network (WM3), inferior parietal WM network (WM4), middle frontal WM network (WM5), anterior cingulum bundle network (WM6), occipital WM network (WM7), orbitofrontal WM network (WM8), middle cingulum bundle network (WM9), precentral/postcentral WM network (WM10), brainstem network (WM11), posterior thalamic radiation and posterior cingulum bundle network (WM12), cerebellum WM network (WM13), and inferior longitudinal fasciculus network (WM14).

### 2.4 Assessment of risk of bias

The risk of bias (RoB) for the non‐randomized studies of interventions (NRSI) was assessed according to Cochrane guideline using the risk of bias in non‐randomized studies of intervention (ROBINS)‐I tool (Sterne et al., 2016). Assessment using ROBINS‐I was conducted on seven domains, including:

(1) Confounding factors, which assess the RoB arising from uncontrolled variables that could affect the outcome; (2) Participant selection for the study, which evaluates how participants are chosen and whether that introduces bias; (3) Intervention classification, which scrutinizes the categorization of interventions; (4) Deviations from intended interventions, which examines whether the interventions were carried out as planned; (5) Missing data, which assesses the impact of incomplete data on the study's conclusions; (6) Outcome measurement, which evaluates the methods used to measure the outcomes of the study; and (7) Selection of reported results, which examines whether the results reported were selectively chosen (Table 3). These domains were categorized into pre‐intervention, intervention and post‐intervention sections. The assessment of each domain, and consequently the overall judgment, is categorized as either low, moderate, serious, critical, and no information. Additionally, the cumulative scores of the RoB for each included study were calculated according to the review authors’ judgments based on various bias domains. These domains were evaluated using a scoring system where Low, Moderate, Serious and Critical values were assigned scores of 1, 2, 3, and 4, respectively. No Information requires a judgment call based on the context of missing information.

**Table 3:** ROBINS‐I risk of bias assessments.

Author	Bias in pre‐intervention		Bias in intervention	Bias in post‐intervention				Result
	due to confounding	in selection of participants for the study	in classi‐fication of interventions	due to deviations from intended interventions	due to missing data	in measurement of outcomes	in selection of the reported result	Overall bias
Ariji et al. (2018)	Serious	Low	Low	Low	Low	Moderate	Low	Serious
Ariji et al. (2019)	Serious	Low	Low	Low	Low	Moderate	Low	Serious
Jin et al. (2021)	Moderate	Low	Low	Low	Low	Moderate	Low	Moderate
Maurer et al. (2021)	Moderate	Low	Low	Low	Low	Moderate	Low	Moderate
Yang et al. (2015)	Serious	Low	Low	Low	Low	Moderate	Low	Serious
Zhang et al. (2021)	Moderate	Low	Low	Low	Low	Moderate	Low	Moderate
Jin et al. (2022)	Moderate	Low	Low	Low	Low	Moderate	Low	Moderate
Zhang et al. (2022)	Moderate	Low	Low	Low	Low	Moderate	Low	Moderate

The assessment of each study for RoB was conducted by two authors (GS and AK) independently. Any differences in opinion were resolved through discussion and consensus and if necessary, with the assistance of further authors (SM or KB).

### 2.5 Assessment of heterogeneity

Assessment of heterogeneity in this SR was conducted qualitatively due to the unavailability of complete quantitative data from all included studies. The evaluation focused on clinical and methodological aspects to gauge the variability across studies. Specifically, the characteristics of each study, the participants involved, the interventions and outcomes were carefully examined.

### 2.6 Assessment of reporting bias

Common reporting biases include publication bias, where studies with positive results are favored; duplicate publication bias, involving redundant publication of the same data; and language bias, which overlooks research published in certain languages, were assessed in the current review (Moher et al., 2003).

### 2.7 Data synthesis and summary measures

The research findings were expounded using a narrative methodology, as the lack of data homogeneity, heterogeneity of included studies and presentation of the results prevented the execution of a meta‐analysis. Consequently, the qualitative exposition of the extracted data focused on conducting comparative assessments among the studies.

This SR involves a comprehensive analysis of the methodologies employed in the included studies to investigate the effects of orthodontic intervention on brain activation. The studies employed fMRI to examine brain regions and networks associated with orthodontic pain. Various techniques, such as BOLD signal measurements, ALFF, and resting‐state FC analyses, were used to assess brain responses and interactions. Demographic information was collected from participants, and clinical assessment such as the Visual Analog Scale (VAS) were employed to assess pain intensity (Tables 1, 4 and Appendix 4: Brain_Regions_Altered_in_Activation_or_FC).

**Table 4:** Assessment of orthodontic pain perception and discomfort.

ID #	Study (author, publication year)	Clinical assessment: time of evaluation mean ± SD
1	Ariji et al. (2018)	**‐VAS values pain/discomfort**: (I) during insertion of the separator: (a) brass contact gauge: 51.8 ± 24.2, (b) floss: 3.3 ± 5.0, *p* = .005 (II) after separator removal (residual pain/discomfort): (a) brass contact gauge: 24.7 ± 25.6, (b) floss: 2.0 ± 2.7, *p* = .008
2	Ariji et al. (2019)	**‐VAS values pain/discomfort**: (I) during insertion of the separator: (a) without biting: 50.1 ± 25.0, (b) with biting: 59.6 ± 26.6 (II) after separator removal (residual pain/discomfort): (a) at rest: 21.0 ± 24.2, (b) with biting: 39.3 ± 30.7, *p* = .0367
3	Jin et al. (2021)	‐ **VAS values pain intensity**: intervention group: before insertion of the separator: 14.7 ± 17.0 with pairing difference: 6.8 ± 16.7, *p* = .010 (before vs after) Control group: 13.7 ± 16.4, *p* = .768 (intervention vs control) ‐ **SCL‐90‐R psychological evaluation**: intervention group: (I) before insertion of the separator 27.7 ± 11.0, *p* = .206 (before vs after) (II) pairing difference: 1.6 ± 8.2 control group: 26.4 ± 11.1, *p* = .573 (intervention vs control)
4	Maurer et al. (2021)	‐ **VAS values pain/discomfort intensity/VAS Anxiety/MPQ** (**NWC, PRI, PRI‐S, PRI‐A, PRI‐E, PRI‐M) pain/discomfort intensity**: (I) before insertion of the separator: 0.2 ± 0.5/0.3 ± 0.6/(2.1 ± 6.2, 4.3 ± 12.9, 1.0 ± 3.0, 0.5 ± 1.6, 0.1 ± 0.3, 0.4 ± 1.3) (II) 24 h after insertion of the separator: 1.0 ± 0.7/0.4 ± 0.7/(5.0 ± 6.0, 10.3 ± 13.0, 2.9 ± 3.1, 0.6 ± 1.6, 0.5 ± 0.5, 1.0 ± 1.3) **‐ STAI‐state/BDI psychological evaluation**: (I) before fMRI and before separator insertion: 35.4 ± 11.7 (II) 24 h after insertion of the separator and before second fMRI: 32.1 ± 12.3 ‐ STAI‐trait: 32.6 ± 9.9, BDI: 3.0 ± 3.9 normal range **‐ WPT/HPT values**: (I) before fMRI and before insertion of the separator: 33.7 ± 0.8°C/43.2 ± 2°C (II) 24 h after insertion of the separator and before second fMRI: 33.9 ± 0.9°C/43.6 ± 0.8°C
5	Yang et al. (2015)	‐ **VAS, PPI, PRI pain intensity**: 24 h after separator insertion and before MRI scan 1.96 ± 1.39, 4.45 ± 2.46, 1.50 ± 0.61
6	Zhang et al. (2021)	‐ **VAS pain intensity**: *p* = .01 (I) before insertion of the separator: 13.66 ± 16.35 (II) 24 h after insertion of the separator and before MRI scan: 20.48 ± 18.09 **‐ SAI questionnaire perception of anxiety**: *p* = .21 (I) before insertion of the separator: 27.73 ± 11.00 (II) 24 h after insertion of the separator and before MRI scan: 29.82 ± 10.48 control group: no measurements of VAS and STAI were obtained.
7	Jin et al. (2022)	‐ **VAS pain intensity**: intervention group: (I) before placement of the separator: 14.7 ± 17.0 (II) 24 h after separator insertion and before MRI scan pairing difference: 6.8 ± 16.7, *t* = −2.7, *p* = .01 (significantly increase in intervention group 24 h after intervention) control group: at baseline: 13.7 ± 16.4, *p* = .768 (intervention vs control) ‐ **SCL‐90‐R psychological evaluation**: intervention group: (I) before placement of the separator: 27.7 ± 11.0 pairing difference: 1.6 ± 8.2, *t* = −1.3, *p* = .206 (no significant differences before and after the intervention) control group: **measurements of VAS and STAI at baseline** 26.4 ± 11.1, *p* = .573 (intervention vs control)
8	Zhang et al. (2022)	**‐ VAS pain intensity**: intervention group: *p* = .018 (1) before insertion of the separator: 14.6 ± 17.3 (II) 24 h after insertion of the separator and before MRI scan: 20.6 ± 17.4 **‐ SAI questionnaire perception of anxiety**: intervention group: *p* = .159 (I) before insertion of the separator: 28.1 ± 11.0 (II) 24 h after insertion of the separator and before MRI scan: 29.9 ± 11.4 **Anxiety and Pain**: Significant positive correlation (*r* = .62, *p* < .001) control group: **no measurements of VAS and STAI were obtained** (before MRI scan, orally confirmation of no pain or discomfort)

Additionally, the studies analyzed correlations between the intensity of orthodontic pain and alterations of brain activation to reveal potential relationships between pain perception and functional interactions among brain regions.

### 2.8 Subgroup and sensitivity analysis

Due to the lack of adequate data, and no possibilities to select homogeneous studies, neither subgroup analyses based on study characteristics nor RoB based sensitivity analyses were performed.

## 3 RESULTS

### 3.1 Study selection

**Figure 1:**
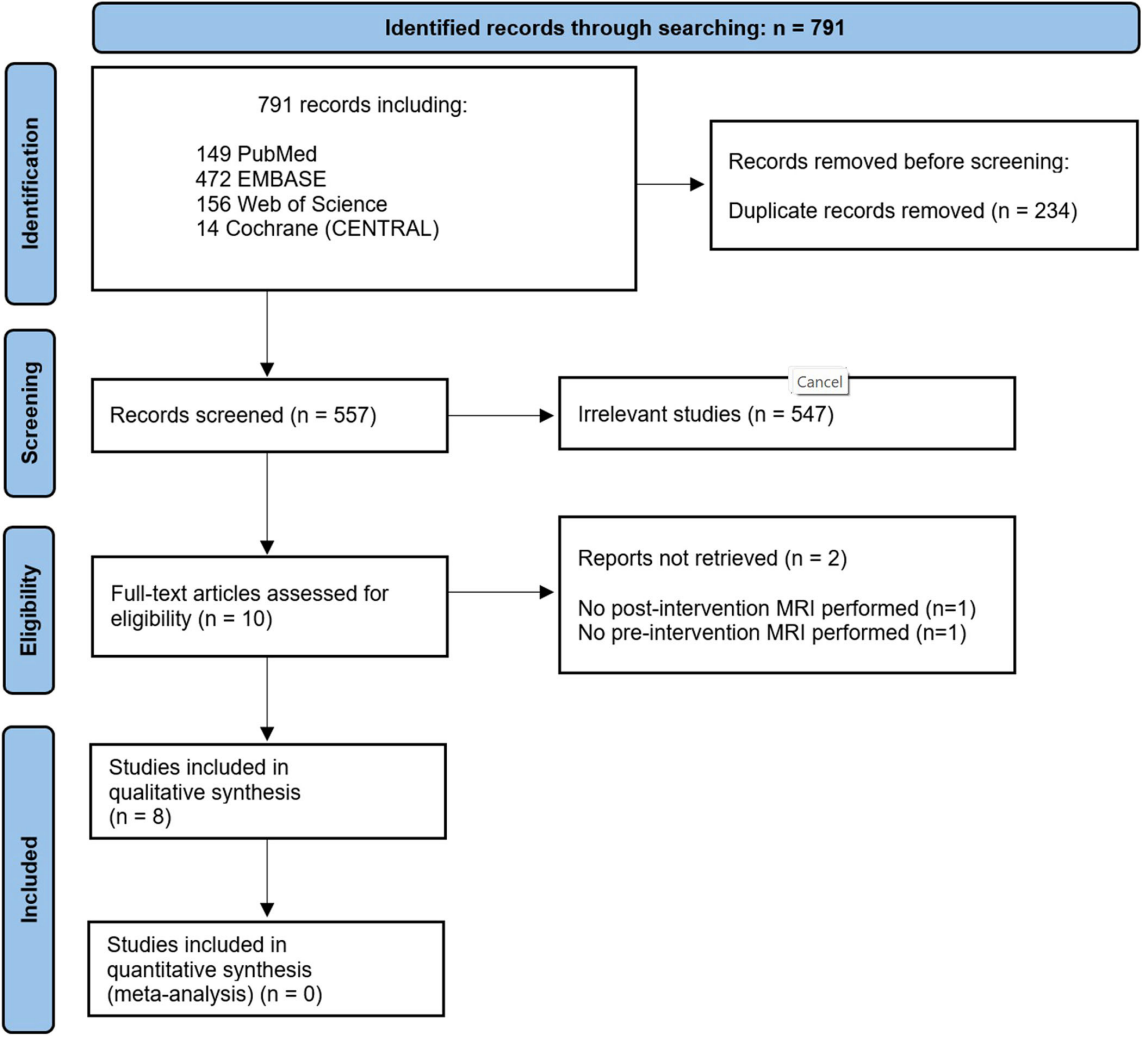
PRISMA flow diagram presenting the search and selection process.

Figure 1 displays the PRISMA flow diagram (Moher et al., 2015) of the literature search process, initially identifying 791 studies from electronic databases. After removing duplicates, 557 publications were screened based on their titles and abstracts. After the eligibility assessment, 10 publications were considered potentially relevant for this SR. However, after a full‐text screening, two articles were excluded (reasons for exclusion are listed in Fig. 1 and Appendix 3_Reasons_of_Exclusion). Finally, eight studies were deemed relevant and met the inclusion criteria for this SR (Ariji et al., 2018; Ariji et al., 2019; Jin et al., 2021; Maurer et al., 2021; Yang et al., 2015; Zhang et al., 2020, Jin et al., 2022, Zhang et al., 2022).

### 3.2 Study characteristics

All studies included in this SR were prospectively designed as NRSI, in accordance with Cochrane guidelines (Sterne & Higgins, 2014). Four of these studies (#1, #2, #4, and #5) did not incorporate a separate control group, whereas the remaining four employed a control group as their comparison (Tables 1 and 2).

### 3.3 Risk of bias in the included studies

In the comprehensive analysis of the selected studies, none were rated as having a low RoB; five exhibited a moderate RoB due to two domains assessed as moderate risk (confounding factors and bias in measurement of outcomes), while the remaining three (#1, #2 and #5) were identified as having a serious risk with one domain (confounding factors) classified as a serious RoB (Table 3).

None of the studies under review provided information about the blinding of selecting participants and operators conducting the assessments, which is a factor in performance bias. As a result, the confounding domain was rated as moderate for the studies included in the review. Blinding is particularly challenging in the evaluation of orthodontic treatment; the applied orthodontic force through an oral appliance is inherently visible and perceptible by both researchers and subjects. However, the use of MRI scans offers an objective measurement. Therefore, the results from those studies using MRI scans are less likely to be influenced by participants' awareness of the intervention they received.

The presence of study‐related bias is acknowledged in this review due to the incorporation of a diverse range of study designs. This decision was made to include as much existing evidence as possible, especially because there are very few studies available on the topic. It is recommended to perform additional clinical trials to foster the establishment of more stringent inclusion criteria. This will potentially pave the way for a meta‐analysis in the future, thereby enhancing the robustness and the comprehensiveness of the evidence.

For the studies reviewed, Ariji et al. (2018 and 2019) and Yang et al. (2015) each had a cumulative score of 10 (according to Table 3 and the Section 2.4.) and indicating the qualitative overall assessment as serious, primarily due to serious concerns in pre‐intervention due to confounding. On the other hand, Jin et al. (2021 and 2022), Maurer et al. (2021), Zhang et al. (2021 and 2022) each recorded a cumulative score of 9 and presenting the moderate level of bias in their qualitative overall assessment. The analysis of the cumulative scores across the studies reveals a mean score of 9.37. Furthermore, the calculated standard deviation, approximately 1.22, suggests a relatively low dispersion of RoB scores around this mean. This indicates that, on average, the studies exhibit a moderate to serious RoB, with variations among them being minimal and tightly clustered around the mean score.

### 3.4 Reporting bias

In the present review, efforts to counteract reporting biases, specifically publication and language biases, were undertaken by initiating a comprehensive and accurate literature search simultaneously across multiple electronic databases without language restrictions. This approach aimed to consider the principles of research integrity and accurate representation of findings.

### 3.5 Heterogeneity and quantitative data synthesis

A quantitative meta‐analysis was not possible to conduct due to heterogeneity. Therefore, the effect estimation could not be evaluated and the qualitative synthesis of each study was assessed separately. The variability in the participants, interventions, and outcomes, such as the properties of the volunteers (age range, gender and sample size) in each study led to the clinical heterogeneity. The diversity in study design, RoB, and the dissimilar intervention components (thickness and hardness of the separators), the varying imaging modalities, and the differences in task and analysis approach caused methodological heterogeneity, which may have contributed to the heterogeneity of the evaluation results.

### 3.6 Qualitative data synthesis

#### 3.6.1 Characteristics of the participants

Given the considerable variability and differences among the selected studies, it would be inappropriate and potentially misleading to aggregate the samples. Therefore, we cannot analyze or combine the participant data from all the included clinical studies collectively. Hence, we observed the participants in the included studies individually. In Studies #3, #5, #6, #7, and #8, a total of five, two, three, five, and four participants were respectively excluded due to head motion that exceeded a predefined threshold during neuroimaging procedures (Table 2). One more participant from #6 was excluded due to potential mental illness. All excluded participants belonged to the intervention groups.

Among the eight studies examined, one study (#4) exclusively included male subjects, while another (#5) solely included female subjects. The remaining two studies (#1 and #2) had a greater proportion of male participants than female, while the other three studies (#3, #6 and #8) had a greater proportion of female participants than male. Study #7 does not specify which part of the sex ratio corresponds to males or females.

Four out of the eight studies (#3, #6, #7 and #8) utilized control groups. The control groups underwent MRI scans without separator placement. In contrast, the remaining four studies did not have separate control groups; two studies (#1 and #2) used baseline measurements for comparison, while two studies (#4 and #5) used pre‐intervention measurements from the same participants in the intervention group 24 h before separator insertion.

Five studies report the participants' age and the average age (#1, #5, #6, #7 and #8), while the other three mention only the mean age (#2, #3, and #4). The minimum age of participants across all studies is 18 and the maximum is 45 and the minimum average age among the studies is 21, and the maximum is 30.8. Three studies had a narrow age range of participants (#5, #6 and #8), while two (#1 and #7) had a broader age range.

#### 3.6.2 Type of the interventions and targeted teeth

Almost all included studies employed orthodontic separator or its alternative (in #1 and #2) to generate mechanical horizontal force to the PDL, which leads to tooth displacement. The separators are generally used to make space between molars and premolars for the implementation of fixed orthodontic devices. In study #1, dental floss with wax and brass contact gauge were used as alternatives to the elastomeric and brass wire separators, respectively. Study #2 used a brass contact gauge as an alternative to the brass wire separator. The utilization of alternative orthodontic appliances in studies #1 and #2 was necessitated by the direct execution of separator insertion within the MRI machine, as the orthodontic plier used for separator insertion was magnetic and could not be introduced into the MRI room. Therefore, a nonmagnetic alternative orthodontic appliance that could be inserted by hand was employed in study #2.

In studies #3 through #8, the duration of the intervention was 24 h. Conversely, studies #1 and #2 employed the intervention duration of 30 and 60 s, respectively.

In five out of the eight included studies (#3, #4, #5, #6, and #7), the separators were inserted into the mandibular molars, whereas studies #1 and #2 targeted the maxillary premolars. Study #8 did not provide anatomical details regarding the location of the treated molars, whether in the mandible or maxilla. In all of the included studies except #3, the teeth on the right side of the jaw were treated.

#### 3.6.3 Assessment of orthodontic pain perception and discomfort

The perception of orthodontic pain is a complex, multidimensional construct influenced by a confluence of sensory discriminative, cognitive affective, and central pathophysiological mechanisms. This subjective experience is modulated by physical and psychological variables, including age, gender, stress, current emotional state, as well as previous pain experiences and the magnitude of the orthodontic force applied (Wiech et al., 2008).

All included studies assessed experimental orthodontic pain and discomfort using VAS. The scales ranged from 0 to 100 in all studies, except for studies #4 and #5, which used scales ranging from 0 to 10. In these scales, 0 represents no pain or no discomfort, while 10 or 100 demonstrates the strongest imaginable pain intensity. Studies #4 and #5 additionally employed the McGill Pain Questionnaire (MPQ), specifically utilizing its components such as the Pain Rating Index (PRI) and the Present Pain Intensity (PPI) (Table 4).

There was a significant increase in pain and discomfort, as measured by the VAS, following the insertion of orthodontic separators in all included studies. However, other psychological and physiological measures like SCL‐90‐R (#3 and #7), Warmth and Heat Pain Thresholds (WPT and HPT) (#4) did not show consistent significant changes. Studies #3, #4 and #5 explored the relationship between pain scores and brain FC alteration. Overall, the insertion of orthodontic separators appears to have a notable impact on perceived pain and discomfort.

The VAS values of pain intensity during separator insertion in Study #1 were 51.8 ± 24.2 for the brass contact gauge and 3.3 ± 5.0 for the floss with a significant difference of *p* = .005 (Wilcoxon rank sum test). VAS values of the residual discomfort directly after separator removal post‐MRI were 24.7 ± 25.6 and 2.0 ± 2.7, respectively. This difference was also significant (*p* = .008).

Study #2 assessed VAS during separator insertion and after its removal. VAS scores indicated higher pain after biting with separator (vertical and horizontal stimulations) compared to rest (without biting, only horizontal stimulation) with the measured values of 59.6 ± 26.6 and 50.1 ± 25.0, respectively. VAS values indicated a residual discomfort of 21.0 ± 24.2 after separator removal at rest and 39.3 ± 30.7 with biting, with a significant difference of *p* = .0367.

Study #3 utilized VAS and SCL‐90‐R for discomfort measurement and psychological evaluation. Both assessments were conducted before the elastic separator placement and also 24 h after insertion before MRI. Before elastic separator placement in the intervention group compared to the control group, there were no significant differences in VAS (14.7 ± 17.0 vs. 13.7 ± 16.4, paired *t*‐test *t* = 0.296) and SCL‐90‐R (27.7 ± 11.0 vs. 26.4 ± 11.1, *t* = 0.566).

In the intervention group, VAS scores increased significantly 24 h after separator insertion compared with that before the elastic separator placement with the pairing difference of 6.8 ± 16.7, *t* = −2.7, *p* = .01 but SCL‐90‐R scores were slightly increased without significant pairing difference of 1.6 ± 8.2, *t* = −1.3, *p* = .206.

In the separator group, significant correlations were identified between the medial thalamus‐seeded FC and VAS scores. Its positive correlations were observed with the right ACC and PCC, whereas a negative correlation was established with the left cerebellum (*p* < .05, AlphaSim correction).

Study #4 assessed pain perception utilizing the MPQ and the VAS for anxiety, pain intensity and discomfort. The mean for anxiety during tooth clenching as a form of vertical stimulation without separator was 0.3 ± 0.6. This increased slightly to 0.4 ± 0.7, 24 h after the separator insertion combined with vertical stimulation. The mean pain intensity and discomfort ratings were also evaluated as 0.05 ± 0.10 and 0.07 ± 0.13 without a separator during tooth clenching, which increased to 1.8 ± 1.70 and 1.05 ± 1.10 post‐separator insertion accompanied with clenching, respectively. Both scales displayed a significant difference between the two examination days (intensity: *z* = −3.4, *p* = .001, *r* = −0.55; discomfort: *z* = −3.4, *p* = .001, *r* = −0.55). In addition to these measures, WPT and HPT were assessed on both examination days before MRI scan. No significant changes were noted in these thresholds. Furthermore, Maurer et al. did not find a significant correlation between VAS scores and alterations in whole brain activities after executing the linear regression analyses.

Study #5 used three scales VAS, PRI and PPI to measure pain intensity 24 h after the placement of orthodontic separator with the values of 1.96 ± 1.39, 4.45 ± 2.46 and 1.50 ± 0.61, respectively. The results showed that there was an increase in pain intensity (PPI and VAS) which had a negative correlation with FC between the postcentral gyrus left and middle temporal gyrus left. No correlation was detected with the PRI scale.

Study #6 assessed pain intensity using a VAS and perception of anxiety employing SAI before and 24 h after inserting an orthodontic elastic separator. Results showed a significant increase in pain intensity 24 h post‐insertion in the separator group (*p* = .01) but no significant change in SAI (*p* = .21) were observed. Additionally, a negative correlation was observed between the VAS pain score and brain FC in specific regions. However, these correlations were not statistically significant after applying the Bonferroni correction for multiple comparisons.

In Study #7 the VAS score in intervention group was measured 14.7 ± 17 before separator insertion, which showed no significant difference to the control group (13.7 ± 16.4, *p* = .768). The paired *t*‐test demonstrated a significant increase in VAS score 24 h after placement of separator in intervention group (6.8 ± 16.7, *t* = −2.7, *p* = .01). Whereas no significant difference was observed in SCL‐90‐R before and after the placement of separators (1.6 ± 8.2, *t* = −1.3, *p* = .206). Additionally, the Pearson correlation analysis and AlphaSim correction for multiple comparisons in the study showed that there was no statistically significant relationship between the fALFF values and the VAS scores.

Study #8 completed the VAS with the value of 14.6 ± 17.3 before the application of the elastic separator and again after 24 h with the value of 20.6 ± 17.4, immediately before MRI scanning. The study found that the pain intensity was significantly higher 24 h after the elastic separator placement (*t* = 2.45, *p* = .018). The study also explored the relationships between topological properties and clinical measurements, including VAS scores. A positive correlation was detected between VAS and STAI scores and nodal efficiency of the right mid‐cingulate cortex (*r *= .303, uncorrected *p* = .040 and *r* = .414, uncorrected *p* = .004, respectively), although these correlations did not survive Bonferroni correction.

#### 3.6.4 Neuroimaging methods

Regarding neuroimaging methods, all included studies utilized fMRI to assess neural activity. Four studies (#3, #5, #6 and #7) employed rs‐fMRI to examine alterations in brain activity after placing an elastic separator through the use of ALFF, fALFF, and FC. FALFF or ALLF was used to examine alterations in spontaneous brain activity in specific regions of the brain, while FC was used to investigate changes in regions of interest (ROIs). By analyzing these changes, rs‐fMRI provided insight into the effects of orthodontic tooth displacement caused by separators on brain activities.

Study #8 utilized rs‐fMRI and graph theory‐based network analysis to explore the organization of the whole brain functional networks.

In contrast, the other included studies (#1, #2 and #4) used task‐based fMRI (t‐fMRI) to investigate regional changes in neural activity during the experimental task, as shown in Table 2.

#### 3.6.5 Findings from the qualitative analyses

Tables 1 and 2 present the demographic characteristics, details of the relevant studies, and the results of neuroimaging analyses. The studies assessed changes in brain activity and FC between various brain regions (see Appendix 4: Brain_Regions_Altered_in_Activation_or_FC). Studies #1, #2, #4 and #7 focused solely on the changes in brain activities. Studies #3 and #5 analyzed both changes in brain activity and FC in ROIs. #6 examined only the alterations in FC, specifically within and between the gray and white matter networks throughout the entire brain and #8 investigated whole‐brain network using global and nodal topological organizations.

In the following section, the outcomes from the individual studies incorporated into this review are explored separately.

‐ #1 (Ariji et al., 2018) utilized fMRI to investigate cerebral area activation shortly after inserting orthodontic tooth separators and indirectly confirmed the possibility of the transmission route from the medulla oblongata to the hypothalamus, providing a potential new therapeutic method to pain and discomfort control. The study found a significant increase in BOLD signals in certain brain regions following the insertion of two types of alternative orthodontic tooth separators compared to baseline. The dental floss with wax and a self‐made brass contact gauge were used as alternatives to elastomeric and brass wire separators, respectively. The separators were inserted between the first and second premolars of the right maxilla in healthy subjects. The insertion took place 60 s after the subjects rested in the MRI machine, and the investigation began 30 s after the apparatus was inserted. The volunteers were randomly assigned to different separators on different days. The outcomes related to brain activity were as follows:

(a) **Comparison of both tooth separators to baseline**:
  A significant increase in BOLD signal following the insertion of dental floss and brass contact gauge in the L parietal association area, L frontal association area, L temporal association area, L insula, and L cerebellum was found. Insertion of the floss increased the BOLD signal in the L hippocampus and L amygdala significantly, whereas insertion of the brass contact gauge increased the BOLD signal in the R thalamus, R hippocampus, R calcarine sulcus, L putamen, and L lingual gyrus significantly.
(b) **Comparison of brass contact gauge with dental floss**:
  Based on the BOLD signals, the comparison showed higher activity in the L thalamus and L cerebellum during brass contact gauge insertion, but no significant differences in other brain regions.

‐ #2 (Ariji et al., 2019) investigated the activated regions in the human brain in response to low‐level clenching and tooth separation, with a focus on identifying differences between the two conditions. The study included two groups of healthy participants: the low‐level clenching group, which performed clenching at two different levels (10% and 40% of the maximum biting force) as vertical stimulation on two separate days randomly, with a 120 s rest period followed by 60 s of clenching; and the tooth separator group, which underwent a biting task that involved both horizontal and vertical stimulation. In the tooth separator group, a brass contact gauge was inserted into the maxillary right premolar, and the biting task was performed at 60, 120, and 180 s after gauge insertion, with 5 s of biting each time. The following main findings of the brain activities were reported:

(a) **Comparison of tooth separator insertion to baseline**:
  The brain regions showing significant activation after the insertion of the brass contact gauge compared to the 60 s baseline included the L primary sensorimotor cortex, L frontal association area, L temporal association area, and both the L and R cerebellum.
(b) **Comparison of tooth separator insertion with biting to rest**:
  The brain regions were activated in the primary sensorimotor cortex, parietal association area, frontal association area, temporal association area, lingual gyrus, thalamus, hippocampus/amygdala, putamen, insula, and cerebellum after insertion with the biting task.
(c) **Comparison of tooth separator insertion with and without biting**:
  The BOLD signals showed a significant increase during 5 s biting, compared to the 60 s baseline after the brass contact gauge insertion (without biting). The significant differences between biting and nonbiting in the separator group were found in the right parietal association area, the right hippocampus/amygdala, and the bilateral parahippocampal gyrus.
(d) **Comparison of biting with tooth separator to low‐level clenching**:
  Hypothalamus was activated in addition to the activated brain regions during low‐level clenching (sensory areas of the cortex, such as the supplementary motor area and primary sensorimotor area).

‐ #3 (Jin et al., 2021) investigated the neural mechanisms of orofacial pain caused by orthodontic elastic separators by analyzing the functions of thalamus as ROI and the FC of two thalamic subregions (medial and dorsal) to other brain regions. The study involved applying elastic separators to the mesial side of the left lower first molar of participants in the intervention group and using an age and sex‐matched control group. The investigation took place 24 h after the separator application:

**Comparison of tooth separator insertion with the control group**:

The subjects in the separator group showed significant alterations in their fALFF and seed‐based FC compared to the control group. The fALFF of the dorsal thalamus was found to be significantly increased, while the fALFF of the medial thalamus was decreased significantly.

Additionally, the FC between the medial thalamus and 12 brain regions (ACC, R parahippocampal gyrus, bilateral middle frontal gyrus, bilateral superior frontal gyrus, R inferior frontal gyrus, R middle temporal gyrus, R insula, and L Thalamus) showed a decrease in activity. The dorsal region of the thalamus was not found to have any alterations in its FC with other brain regions.

‐ #4 (Maurer et al., 2021) utilized t‐fMRI to examine the significant activation of brain regions 24 h after the insertion of an elastic separator between the right mandibular second bicuspid and first molar, combined with tooth clenching (painful tooth clenching). The study compared this task with the same participants who only clenched their teeth without the separator. The participants performed tooth clenching 36 times per event, with each clenching lasting 3 s and separated by rest periods of 20‐30 s. The results of the two comparisons are presented as follows:

(a) **Comparison of painful tooth clenching to rest**:
  The brain regions were activated significantly during painful tooth clenching in the bilateral anterior and posterior insula, bilateral thalamus, the bilateral secondary somatosensory cortex (S2), bilateral inferior frontal gyrus (IFG), bilateral putamen, bilateral inferior parietal lobule (IPL), middle cingulate cortex (MCC), bilateral middle frontal gyrus (MFG), bilateral superior frontal gyrus (SFG), bilateral cerebellum, and the left primary motor cortex (M1).
(b) **Comparison of tooth clenching with separator to experimental tooth clenching**:
  The comparison between the BOLD response during painful tooth clenching and tooth clenching without a separator revealed increased activations in several brain regions in the L primary somatosensory cortex (S1), S2, M1, supplementary motor area (SMA), right rolandic operculum, and bilateral insula (both anterior and posterior).

‐ #5 (Yang et al., 2015) utilized rs‐fMRI to examine the changes in brain activity that occurred as a result of the placement of an elastic separator between the mesial and distal sides of the right mandibular first molar by measuring the BOLD signals. This study employed ALFF analysis in brain regions and FC analysis in the ROIs. The scans were performed before and 24 h after the insertion of the elastic separator resulting as follows:

**Comparison before and after separator insertion**:
The comparison of ALFF values between subjects in the normal and intervention conditions showed increased activity in the L insular cortex (IC.L) in the sub‐lobar region and the SMA.R in the frontal lobe. Conversely, a decrease in ALFF was observed in the L pyramis and R uvula in the bilateral cerebellar posterior lobe, the R angular gyrus in the parietal lobe, the L angular gyrus in the precuneus, and the L superior frontal gyrus in the frontal lobe.

The comparison of FC changes based on ROIs after and before separator insertion revealed an increase in FC in the pyramis of the L cerebellum posterior lobe (CPLP.L) (ROI: R parietal lobe). The decrease in FC was detected in the pyramis of the R cerebellum posterior lobe (CPLP.R) (ROI: L insular cortex), L middle temporal gyrus (MTG.L) (ROI: L precuneus), L parietal lobe (PL.L), and cuneus occipital lobe (COL) (ROI: L posterior cerebellum).

‐ #6 (Zhang et al., 2022) analyzed rs‐fMRI data and found significant alterations in FC within and between 12 gray matter (GM) and 14 white matter (WM) networks, as well as in three loops, 24 h after insertion of an elastic separator between the first and second molars on the right side of the mandible, compared to sex‐ and age‐matched healthy control group.

(I) In the GM networks, the study found increased FC between the DAN, DMN and SN. Additionally, the study identified decreased FC between the anterior cerebellum lobe network, DAN, and ECN.

(II) In the WM networks, increased FC was observed between the posterior thalamic radiation and posterior cingulum bundle network (WM12) with the following WM networks: WM1, WM4 and WM14. Decreased FC was detected in two pathways: (1) WM1‐WM2‐WM11‐WM3‐WM4‐WM5‐WM11 and (2) WM5‐WM12‐WM9.

(III) Between GM and WM networks, increased FC was investigated in: GM5‐WM4, GM6‐WM9‐GM7‐WM2 and between WM12 and GM3, GM4, GM5, GM6, GM7, GM8, GM9, GM11, GM12 networks.

Furthermore, FC was decreased in: GM3‐WM5, GM8‐WM11, and WM1‐GM10‐WM2.

(IV) Alterations in three GM‐WM‐loops was identified as following: (1) DMN‐WM12‐WM4‐DMN, (2) DAN‐WM12‐WM5‐DAN and (3) SN‐WM12‐WM9‐SN.

‐ #7 (Jin et al., 2022) employed rs‐fMRI to investigate the alterations in intrinsic cerebral activity induced by orthodontic separator and utilized fALFF metrics to assess regional brain functions. The MRI scans were conducted before and 24 h after the insertion of the elastic separator with the following outcomes:

**Comparison of tooth separator group to the healthy controls**:
The fALFF analysis revealed that, relative to the control group, the tooth separator group demonstrated increased activity in the L cerebellum, R posterior cingulate gyrus, and bilateral inferior temporal gyrus. Conversely, decreased fALFF was noted in the medial prefrontal cortex, L ACC, bilateral angular gyrus, L inferior parietal cortex, middle temporal gyrus, and additional miscellaneous cerebral regions. The study concluded that these aberrant functional activities were predominantly localized within the DMN.

‐ #8 (Zhang et al., 2022) utilized graph‐theoretical network analyses on rs‐fMRI data to investigate the neural underpinnings of orthodontic pain induced by elastic separator placement for 24 h in comparison to a control cohort with the following results:

**Comparison of tooth separator group to the healthy controls**:
The impact of an elastic separator on brain network topology was examined in this clinical trial in comparison to a control group. Global topological metrics showed a decrease in both clustering coefficient and local efficiency, indicating compromised network integrity. Nodal topology revealed increased centrality in ipsilateral brain regions and decreased centrality contralaterally. Correlation and mediation analyses linked nodal efficiency in the R mid‐cingulate cortex to clinical measures of pain (VAS) and anxiety (SAI) at 24 h post‐intervention, although these correlations were not statistically significant.

## 4 DISCUSSION

### 4.1 Overview of findings

The aim of this SR was to evaluate the effect of orthodontic tooth displacement on the CNS in humans using fMRI which were considered by the literature search. All of the included studies utilized orthodontic separators as the intervention but they did not confirm whether they moved teeth. However, in a previous study, it has been confirmed that the placement of orthodontic separator produces tooth displacement within the periodontal space, even short time after its insertion (Asiry, 2018; Davidovitch et al., 2008). This initial phase of OTM, often associated with patient‐reported pain, is a crucial area of study in OTM. This review demonstrates the complex interactions between orthodontic treatment, pain perception, and brain function and provides insights into potential strategies to minimize discomfort and pain during orthodontic treatment in future research.

The biological aspect of OTM is due to bone turnover, which is regulated by the sympathetic nervous system and its associated pathway in the human brain (Ariji et al., 2019). The sympathetic nervous system influences osteoblast and osteoclast activities (Corr et al., 2017). Animal studies have shown that orthodontic appliances induce osteoclast activation and increase the sympathetic neuromarker, around the tooth root (Kondo et al., 2013). In contrast, mice with denervated sympathetic nervous systems showed no such increases after inserting orthodontic appliances (Cherruau et al., 2003; Corr et al., 2017). Moreover, hypothalamus destruction in mice prevents osteoclast activity elevation in periodontal tissues (Oheim et al., 2013). Study #1 indirectly confirmed the possibility of the transmission route from the medulla oblongata to the ventromedial nucleus of the hypothalamus in human via increasing the BOLD signals, which results in sympathetic nervous system activation. The use of nonsteroidal anti‐inflammatory drugs (NSAIDs) to inhibit prostaglandin synthesis and reduce inflammation to the PDL provide pain relief during orthodontic treatment. However, these drugs may delay the rate of tooth movement (Walker & Buring, 2001) and hinder the optimal progression of treatment. Orthodontic tooth separation initiates afferent signals that are interpreted as pain by the CNS. Following this, these signals elicit efferent signals that modulate bone metabolism within the periodontal structures (Cherruau et al., 2003), through the sympathetic nervous system under the governance of the hypothalamic area which was confirmed by Study #2. These findings provide support for the hypothesis that an agonist of the sympathetic nervous system may be considered as a potential strategy for mitigating orthodontic pain without adversely affecting OTM. This could have the ability to stimulate the release of endorphins and other pain‐relieving substances within the body, thereby effectively alleviating pain.

In studies #2 and #4, the intervention was employed accompanied with experimental biting or clenching to strengthen pain typically experienced by patients undergoing orthodontic treatment. Both studies firstly observed the effects of biting or clenching on brain without the use of separators. This was performed to isolate and evaluate the role of separators when it is later employed in conjunction with biting. Understanding the impact of this everyday activity is essential for both future interpreting the research accurately and applying it effectively in orthodontic treatment.

In the included studies, participants demonstrated changes in brain activities and FC after the placement of separators. These changes occurred primarily in brain regions associated with the pain matrix and the limbic system, as extensively reported.

Despite the heterogeneity among the included studies regarding their methodologies and clinical aspects, they collectively indicate that, in the early phase of orthodontic treatment, specific brain areas undergo changes in activation and their FC.

### 4.2 Alteration in regional brain activity

In our included studies the thalamus, insula, frontal area, and cerebellum were most mentioned in alterations of brain regions' activity, indicating their significant role in the pain matrix (Davidovitch et al., 2008).

**‐ Thalamus**: The thalamus is the main component of the pain matrix and is activated to perceive orthodontic pain and subjected to pain modulation through the involvement of its medial and dorsal subregions (Corr et al., 2017; Kondo et al., 2013) The medial thalamus, closely connected to the limbic system, is likely to play a vital role in the cognitive and emotional modulation of orofacial pain (Jin et al., 2021). Study #3 identified decreased fALFF in the medial and increased fALFF in the dorsal area of the thalamus after the insertion of the orthodontic separator. The ventroposterior nucleus in the dorsal thalamus transmits nociceptive information to the cortex and may be involved in sensory discrimination (Groh et al., 2018; Long et al., 2016) A decrease in activity of the medial thalamus might suggest reduced emotional engagement or affective response to the pain while an increase in activity in the dorsal thalamus could imply heightened sensory awareness or perception of the pain.

The significant increase in BOLD signals in the L thalamus following the insertion of a brass contact gauge, as compared to floss (#1), indicated that higher levels of pain from the brass contact gauge influenced thalamic activation. The dental floss caused only minor pain, leaving the question whether it serves as an adequate alternative to the elastomeric separator.

Additionally, painful tooth biting/clenching with a separator (#2 and #4) resulted in significantly higher BOLD signals in the thalamus likely attributed to the greater pain response experienced during the task and increased discomfort reported after examination. This result suggests that the relationship between dental stimulation and thalamus activity may be linked to the intensity of the painful stimulus.

**‐ Insula**: Most of the included studies showed increased BOLD signals in the insula after separator insertion. The anterior and posterior insular regions were found to have stronger activity bilaterally during painful tooth clenching (#4). The insular cortex (IC) is an affective component of pain perception and is involved in the emotional experience of pain relevant to memory (Fulbright et al., 2001). Many studies showed that the reaction of the front part of IC to painful stimuli diminishes over time (Fantozzi et al., 2019). This indicates that a reduced functional activity of the front part of the insular cortex can result in decreased pain memory in pain experiences (Andreasen et al., 1999; Yang et al., 2015).

**‐ Frontal area**: In the individuals with orthodontic separators, the frontal association area, which is involved in cognition and judgment, showed increased activity (#1 and #2). Additionally, in the subjects with experimental painful tooth clenching compared to the subjects with orthodontic separator, BOLD signals showed significantly increased activation in the bilateral inferior frontal gyrus, bilateral middle frontal gyrus, and bilateral superior frontal gyrus (#4). Study #8 observed frequently heightened functional activity in frontal gyrus. While #5 showed significant decrease in ALFF signals in the superior frontal gyrus after insertion of the orthodontic separator. This divergence in findings could be attributed to clinical and methodological heterogeneities in the studies.

**‐ Cerebellum**: Most of the included studies have consistently highlighted a notable rise in activity within the cerebellum. Traditionally recognized for its contributions to motor control and cognitive processing, the cerebellum also takes part in modulating sensory experiences and retrieving episodic memories (Fantozzi et al., 2019; Fulbright et al., 2001). These functions hint at the cerebellum's possible role in managing pain.

**‐ Limbic system**: The alteration in activation of the parts of the limbic system, including the ACC, prefrontal cortex (PFC), insula, temporal cortex, thalamus, middle cingulate gyrus, parahippocampal gyrus, amygdala, and hippocampus, was observed in the most of the included studies. These brain regions work together to create emotions, memories, and behavior (Aggleton et al., 1995; Torrico & Abdijadid, 2022). Altered activation of the limbic system during orthodontic procedures could potentially affect how the experience of pain is encoded into memory, influencing future reactions to similar treatments or stimuli. Understanding these neural correlates could have benefits, both for improving patient care and for advancing our understanding of neural responses to pain.

**‐ Temporal area**: Study #7 observed a decrease in fALFF in the Medial Temporal Lobe (MTL), an area essential for memory formation and mental simulation. This observation is particularly relevant given that dental patients frequently recall more pain than initially reported during procedures, a trend accentuated in those with dental fear (Kyle et al., 2016). The decreased activity in the MTL may be linked to its role in encoding memories of orthodontic pain, although additional studies are required for confirmation.

Notably, the parahippocampal cortex within the MTL is essential for recognition and source memory. This observation is supported by studies #1 and #2, which also reported elevated activity in the temporal association area.

**‐ Other Regions**: The hippocampus plays a crucial role in forming and retrieving long‐term memories (Kesner & Rolls, 2015) and is vulnerable to neurological and psychiatric conditions such as Alzheimer's disease (Eichenbaum and Cohen, 2014; Eichenbaum, 2004), which means increasing hippocampal perfusion can influence spatial memory (Houk et al., 2010; Voss et al., 2013). Study #1 found increased BOLD signals in the hippocampus and amygdala in the subjects with orthodontic separator. Study #6 suggests that pain signals can be transmitted via thalamic radiation to the amygdala, hippocampus, and other brain regions involved in pain processing. The amygdala is essential for processing emotional information and forming emotional memories, while the ACC and insula encode the emotional aspects of pain (Aggleton et al., 1995).

### 4.3 Alterations of functional connectivity

Three of the eight included studies investigated the alteration of FC: Studies #3 and #5 explored the FC in ROI, while study #6 observed the whole‐brain FC, investigating the FC within and between GM and WM networks and loops. Study #8 also investigated alterations in FC, focusing on the impact of experimental orthodontic tooth displacement on global and local changes in brain functional network topology. There are several common results regarding FC and its influences on pain perception, cognition, and emotion:

**‐ Network interactions**: Study #6 specifically highlights changes in FC between various GM networks, including the Dorsal Attention Network (DAN), Default Mode Network (DMN), and Salience Network (SN). The disruption of network interactions caused by orthodontic procedures may lead to changes in attention, cognitive processes, and emotion regulation. For example, the DAN is involved in attentional control and directing focus, so alterations in its connectivity may affect an individual's ability to concentrate or maintain attention (Yeager et al., 2021). The DMN, on the other hand, is associated with self‐reflection, introspection, and mind‐wandering (Zhou & Lei, 2018). Changes in its connectivity could influence self‐awareness or the ability to engage in internal mental processes. Study #7 observed notable changes in activity in key nodes of the DMN, specifically the PCC, medial PFC, inferior parietal cortex, and angular gyrus. Furthermore, study #5 suggested that separator insertion might exert a transient inhibitory effect on DMN functionality. Additional investigations into the DMN as a highly stable network and the key role in the processing of orthodontic pain are still required. Moreover, the SN plays an essential role in detecting and integrating salient sensory information and coordinating appropriate responses. Disrupted connectivity within this network may influence the processing of sensory stimuli and the regulation of emotional responses.

**‐ Cerebellar involvement**: Studies #5 and #6 identify FC changes within cerebellar networks, particularly in the cerebellum posterior lobe, which is essential for motor control and coordination. The pyramis in the cerebellar vermis, plays a significant role in motor movements, perception, cognition, and attention. These studies, along with Study #3, provide evidence for the modulation of pain perception by demonstrating connectivity between the cerebellum and the thalamic area. These findings highlight the potential significance of the cerebellum in the sensory and cognitive aspects of perceiving orthodontic pain and its role in pain modulation.

**‐ Pain perception and cognition**: The thalamus as the vital part of the pain matrix was observed in most of the FC alterations. Study #3 reported a decrease in FC between the medial thalamus and various brain regions, including the cerebellum, ACC, parahippocampal gyrus, frontal gyrus, temporal gyrus, and insula. These changes in FC suggest the involvement of the medial thalamus in the cognitive and emotional modulation of orofacial pain. Furthermore, study #6 found increased connectivity between WM12 network (which includes posterior thalamic radiation and posterior cingulum bundle) and the most GM networks. Additionally, significant alterations in FC were observed in the WM12 network, specifically in relation to three GM‐WM loops. These loops involved DMN, DAN and SN. These findings demonstrate that the participants with orthodontic tooth separators exhibit significant changes in FC within networks associated with pain processing. These alterations may be influenced by a WM network related to emotion perception and cognitive processing.

**‐ Other observations**:

Study #6 highlights decreased FC between the executive control network (ECN) and the brainstem network, suggesting a disruption in cognitive control processes. These findings imply that orthodontic procedures may affect cognitive functions related to self‐regulation and decision‐making.

Study #8 mainly observed the alteration nodal centrality, which can be interpreted as changes in activation or connectivity in ipsilateral brain nodes (right side) and contralateral brain nodes (left side) and concluded an increase in mainly ipsilateral brain areas and a decrease in contralateral brain nodes. In this study, the right MCC was found to have a significant role in the context of orthodontic pain.

However, it is important to note that these conclusions are specific to the included studies in this SR. Further research is needed to fully understand the implications and functional significance of these FC alterations in different contexts or populations.

### 4.4 Limitations of included studies

In this review, several limitations were encountered which could potentially impact the interpretation and generalizability of the findings. Firstly, the review noted a lack of randomized controlled trials, which are generally considered to yield more reliable results. This absence is reflective of the limited research available in this specific field, as only non‐randomized studies were identified during the database search. The included studies comprised clinical trials with and without control groups. Given the limited number of studies included, it was imperative to assess and evaluate both types of studies collectively. This introduced heterogeneity and could potentially affect the synthesis and interpretation of the findings.

Additionally, the review was limited by the exclusive use of specific medical databases and the strict adherence to the predefined inclusion and exclusion criteria. These factors could have restricted the breadth of evidence retrieved and analyzed.

The covariates mentioned in this review (Table 1) can be considered as another limitation due to their effect on the results and interpretation of the findings. These factors can potentially affect brain structure and function and should be taken into consideration when analyzing neuroimaging data related to orthodontic treatment or pain. The sensation of induced orofacial pain depends upon several factors, such as gender, emotional state, social state, handedness, the magnitude of applied orthodontic force, and other physical and psychological factors (Marini et al., 2013), which are not all considered the same in all included studies.

The limited sample sizes in the included studies reduce the statistical power, thereby affecting the reproducibility and generalizability of the outcomes. Future research should aim to include larger sample sizes.

To investigate the pure assessment of the intervention's effect, we restricted our analysis to studies utilizing healthy volunteers as subjects, rather than including studies that focus on clinical patient populations in real‐world healthcare situation. Future studies can be conducted to examine the patients with an indication for orthodontic treatment to better investigate the mechanism of the effects of OTM in the patients' daily lives.

All included studies concentrated on short‐term experiments immediately (#1 and #2) to 24 h (6 remaining studies) after placement of separators in the initial phase of tooth movement. The results may not fully be applicable to long‐term orthodontic treatments, in which patients get used to the treatment overtime. In the framework of this SR, it was initially expected to investigate the effects of orthodontic tooth displacement caused by both fixed and removable orthodontic appliances on brain activation. However, after conducting the literature search, all included studies pertained solely to removable separators.

Two of the included studies examined the ROI (#3 and #5) instead of whole‐brain analysis, which may represent another limitation in this review. ROI studies focused on predefined brain regions, which limited the generalizability of the findings to the entire brain. Different ROIs in different studies can also lead to inconsistent results and difficulties in comparing the results between the studies.

## 5 CONCLUSIONS AND FURTHER CONSIDERATIONS

The included studies in this SR revealed changes in brain activity and FC between brain regions, including not only the pain matrix but also other regions involved in cognition, memory and emotion (limbic system) post‐separator insertion. However, due to the limited number of participants in the included studies, the varying types of studies, and the limitations noted, additional research is necessary to obtain more robust and reliable results.

Further studies may also consider exploring gender differences and the potential impact of sex hormones on the pathophysiology of pain in male and female participants with larger sample sizes. Due to the limitations of the included studies, further clinical studies are recommended to longitudinally investigate the long‐term effect of orthodontic treatment on brain activation.

Moreover, understanding the specific brain regions involved in the processing of experimental tooth displacement during the early phase of orthodontic treatment can aid in the development of targeted treatments that focus on the underlying mechanisms of pain. This could make orthodontic treatment more comfortable and prevent patients from abandoning the treatment, which often happens at the start of the orthodontic process. By identifying the brain regions involved in orthodontic pain perception, the results could also contribute to the development of treatments that reduce the perception of pain without adversely affecting the procedures essential for tooth movement. Nevertheless, this SR paves the way for future clinical research by addressing the heterogeneity and limitations encountered in this study, thereby fostering a more streamlined and robust investigative framework for subsequent inquiries.

## AUTHOR CONTRIBUTION

Gelareh Sadvandi: Conceptualization and design, Methodology: Search strategy, selection of studies, data collection and analysis according to the PICO format, formal analysis, investigation, original draft preparation and writing the manuscript, editing and revision, visualization. Amir Ehsan Kianfar: Search strategy, selection of studies, data collection and analysis according to the PICO format, formal analysis, (all independently), critical Review. Kathrin Becker: Supervision, critical review and correcting. Michael Wolf: Critical review. Alexander Heinzel: Critical review and correcting. Sareh Said‐Yekta Michael: Conceptualization and design, supervision, critical review and correcting, correspondence. All authors reached consensus on the content, submission, and publication of this review.

## CONFLICT OF INTEREST STATEMENT

The authors have stated explicitly that there are no conflicts of interest in connection with this article.

## ETHICAL STATEMENTS

Ethical approval for this study is not necessary since it exclusively utilizes data from prior published research. Each included study in this review has undergone and received approval from their respective ethical committees.

## Supporting information

Supplementary information.

Supplementary information.

Supplementary information.

Supplementary information.

## Data Availability

All data supporting the findings of this systematic review are available within the paper and its supplementary material.
