# Supplementary material for: Systematic review on effects of experimental orthodontic tooth displacement on brain activation assessed by fMRI
Source: Clin Exp Dent Res. 2024 Apr 1;10(2):e879. doi: 10.1002/cre2.879 (PMC10982672; doi:10.1002/cre2.879)
Supplement: Supplementary file 2 — Supplementary information. [file CRE2-10-e879-s003.docx]

**Appendix 3: Reasons of Exclusion**

**Table 1: Reasons for exclusion after full-text screening**

Total number of full-text articles excluded: n = 2

| **No** | **Study** | **Reason for exclusion** |
| --- | --- | --- |
| 1 | Ayoub et al. 2021, Baseline resting-state functional connectivity determines subsequent pain ratings to a tonic ecologically valid experimental model of orofacial pain. PAIN 162(9): P 2397-2404, September 2021. DOI: 10.1097/j.pain.0000000000002225 | No post-intervention MRI performed |
| 2 | Yang et al. Placebo modulation in orthodontic pain: a single-blind functional magnetic resonance study. Radiol med 126, P 1356–1365 October 2021. DOI: 10.1007/s11547-021-01374-4 | No pre-intervention MRI performed |

**Table 2: Exclusion by title and abstract screening**

Total number of articles excluded after duplicate removal: n = 547

| **No** | **Study** | **DOI/ PMID** | **Author** |
| --- | --- | --- | --- |
| 1 | Imaging the neural substrate of trigeminal neuralgia pain using deep learning. | 10.3389/fnhum.2023.1144159 | Liang 2023 |
| 2 | The genetic spectrum of congenital ocular motor apraxia type Cogan: an observational study, continued. | 10.1186/s13023-023-02706-5 | Schröder 2023 |
| 3 | The effect of metallic dental restorations and implants in causing patient discomfort and artefacts during magnetic resonance imaging of the head and neck. | 10.4103/ijdr.ijdr_430_21 | Ashok 2022 |
| 4 | Natural History of SURF1 Deficiency: A Retrospective Chart Review. | 10.1016/j.pediatrneurol.2022.12.002 | Khan 2023 |
| 5 | Variances of quantifying of Virchow-Robin spaces detecting the different functional status of glymphatic system in simple febrile seizures affected by seizures duration. | 10.1097/MD.0000000000032606 | Li 2022 |
| 6 | Children with autism spectrum disorder present glymphatic system dysfunction evidenced by diffusion tensor imaging along the perivascular space. | 10.1097/MD.0000000000032061 | Li 2022 |
| 7 | Editorial for "Preoperative MRI-Based Radiomics of Brain Metastasis to Assess T790M Resistance Mutation After EGFR-TKI Treatment in NSCLC". | 10.1002/jmri.28444 | Chaurasia 2023 |
| 8 | Long-Surviving Adult Siblings With Joubert Syndrome Harboring a Novel Compound Heterozygous CPLANE1 Variant. | 10.1212/NXG.0000000000200031 | Matoba 2022 |
| 9 | Cerebral control of swallowing: An update on neurobehavioral evidence. | 10.1016/j.jns.2022.120434 | Cheng 2022 |
| 10 | Joubert Syndrome with a Rare Ocular Phenotype: Coloboma with Retrobulbar Cysts - A Case Report. | 10.1159/000525798 | Chettiankandi 2022 |
| 11 | Magnetoencephalographic evaluation of repaired lip sensation in patients with cleft lip. | 10.1371/journal.pone.0274405 | Kitayama 2022 |
| 12 | Disrupted Spontaneous Neural Activity and Its Interaction With Pain and Emotion in Temporomandibular Disorders. | 10.3389/fnins.2022.941244 | Chen 2022 |
| 13 | Validation of Muscle Fiber Architecture of the Human Tongue Revealed by Diffusion Magnetic Resonance Imaging With Histology Verification. | 10.1044/2022_JSLHR-22-00040 | Liang 2022 |
| 14 | Sleep, Respiration and Nocturnal Paroxysmal Events in Joubert Syndrome: A Case Report. | 10.2147/NSS.S369097 | Peraita-Adrados 2022 |
| 15 | Primary intracranial extraskeletal myxoid chondrosarcoma: A case report and review of literature. | 10.12998/wjcc.v10.i13.4301 | Zhu 2022 |
| 16 | Artifacts in magnetic resonance imaging caused by dental materials: a systematic review. | 10.1259/dmfr.20210450 | Bohner 2022 |
| 17 | Systematic analysis of physical examination characteristics of 94 individuals with Joubert syndrome: Keys to suspecting the diagnosis. | 10.1002/ajmg.c.31966 | Forsyth 2022 |
| 18 | Joubert Syndrome Presenting With Oculomotor Apraxia and Motor Developmental Delay: A Case Report From a Neuro-Ophthalmology Clinic in Saudi Arabia. | 10.7759/cureus.21638 | Mandura 2022 |
| 19 | Functional Changes in Brain Activity Using Hypnosis: A Systematic Review. | 10.3390/brainsci12010108 | Wolf 2022 |
| 20 | A clinically applicable functional MRI memory paradigm for use with pediatric patients. | 10.1016/j.yebeh.2021.108461 | Shurtleff 2022 |
| 21 | Abnormal static and dynamic brain function in patients with temporomandibular disorders: a resting-state functional magnetic resonance imaging study. | 10.7518/hxkq.2021.06.009 | Yin 2021 |
| 22 | Kernicterus Spectrum Disorders Diagnostic Toolkit: validation using retrospective chart review. | 10.1038/s41390-021-01755-5 | Dasari 2022 |
| 23 | Magnetic resonance imaging of brain anomalies in adult and pediatric schizophrenia patients: Experience of a Romanian tertiary hospital. | 10.3892/etm.2021.10532 | Iliuta 2021 |
| 24 | Homozygosity for a novel missense variant of RPGRIP1L causing Joubert syndrome with renal defects in a family of Chinese descent. | 10.5414/CN110539 | Sun 2021 |
| 25 | How Memory Switches Brain Responses of Patients with Post-traumatic Stress Disorder. | 10.1093/texcom/tgab021 | Inoue 2021 |
| 26 | Preauricular Intraparotid Schwannoma: A Rare Presentation with Literature Review. | 10.4103/ccd.ccd_519_20 | Bagga 2021 |
| 27 | Two cases of DYNC1H1 mutations with intractable epilepsy. | 10.1016/j.braindev.2021.05.005 | Matsumoto 2021 |
| 28 | A Resting-state Functional Magnetic Resonance Imaging Study of Whole-brain Functional Connectivity of Voxel Levels in Patients With Irritable Bowel Syndrome With Depressive Symptoms. | 10.5056/jnm20209 | Li 2021 |
| 29 | Prospective motion detection and re-acquisition in diffusion MRI using a phase image-based method-Application to brain and tongue imaging. | 10.1002/mrm.28729 | Liang 2021 |
| 30 | A de novo frameshift variant of ANKRD11 (c.1366_1367dup) in a Chinese patient with KBG syndrome. | 10.1186/s12920-021-00920-3 | Chen 2021 |
| 31 | Repeated cerebellar infarction in the affected nondominant vertebral artery distribution with reversible vertebral artery occlusion elicited by head tilt: illustrative case. | 10.3171/CASE2061 | Nozawa 2021 |
| 32 | The neuro-pathophysiology of temporomandibular disorders-related pain: a systematic review of structural and functional MRI studies. | 10.1186/s10194-020-01131-4 | Yin 2020 |
| 33 | Preliminary evaluation of pre-speech and neurodevelopmental measures in 7-11-week-old infants with isolated oral clefts. | 10.1038/s41390-020-0887-5 | Conrad 2021 |
| 34 | Co-occurrence of mutations in KIF7 and KIAA0556 in Joubert syndrome with ocular coloboma, pituitary malformation and growth hormone deficiency: a case report and literature review. | 10.1186/s12887-020-2019-0 | Niceta 2020 |
| 35 | Relationship between vertical facial pattern and brain structure and shape. | 10.1007/s00784-020-03227-2 | AlarcÃ³n 2020 |
| 36 | Novel compound heterozygous TMEM67 variants in a Vietnamese family with Joubert syndrome: a case report. | 10.1186/s12881-020-0962-0 | BuiTPH 2020 |
| 37 | PQBP1, an intellectual disability causative gene, affects bone development and growth. | 10.1016/j.bbrc.2019.12.097 | Yang 2020 |
| 38 | Magnetic Resonance Imaging and Its Effects on Metallic Brackets and Wires: Does It Alter the Temperature and Bonding Efficacy of Orthodontic Devices? | 10.3390/ma12233971 | Sfondrini 2019 |
| 39 | Performing Diffusion Tensor and Functional MRI in Patients with Metallic Braces. | 10.1148/radiol.2019192297 | Dietrich 2020 |
| 40 | Whole-Brain Functional and Diffusion Tensor MRI in Human Participants with Metallic Orthodontic Braces. | 10.1148/radiol.2019190070 | Miao 2020 |
| 41 | Data on MRI brain lesion segmentation using K-means and Gaussian Mixture Model-Expectation Maximization. | 10.1016/j.dib.2019.104628 | Qiao 2019 |
| 42 | Evaluation of temporomandibular joint, masticatory muscle, and brain cortex activity in patients treated by removable functional appliances: a prospective fMRI study. | 10.1259/dmfr.20190216 | Ozdiler 2019 |
| 43 | [Relationship between masticatory muscles activity and motor cortex activation during treatment of patients with distal malocclusion]. | 10.17116/stomat20199803171 | Markov 2019 |
| 44 | Unbalanced Occlusion Modifies the Pattern of Brain Activity During Execution of a Finger to Thumb Motor Task. | 10.3389/fnins.2019.00499 | TramontiFantozzi 2019 |
| 45 | [Advances in application of functional magnetic resonance imaging in patients with painful temporomandibular disorders]. | 10.3760/cma.j.issn.  1002-0098.2019.05.011 | Yin 2019 |
| 46 | Comparison of 2D BLADE Turbo Gradient- and Spin-Echo and 2D Spin-Echo Echo-Planar Diffusion-Weighted Brain MRI at 3 T: Preliminary Experience in Children. | 10.1016/j.acra.2019.02.002 | Hu 2019 |
| 47 | Review of Ocular Manifestations of Joubert Syndrome. | 10.3390/genes9120605 | Wang 2018 |
| 48 | Nystagmus And Beyond: A Rare Ocular Motility Disorder. | PMID: 30465390 | Naqaish 2018 |
| 49 | A Novel Filtering Approach for 3D Harmonic Phase Analysis of Tagged MRI. | 10.1117/12.2293643 | Wang 2018 |
| 50 | MRI safety: MRI and fixed orthodontic appliances. | 10.1038/sj.bdj.2018.935 | Stonier 2018 |
| 51 | Correcting B(0) Field Distortions in MRI Caused by Stainless Steel Orthodontic Appliances at 1.5 T Using Permanent Magnets - A Head Phantom Study. | 10.1038/s41598-018-23890-6 | Wang 2018 |
| 52 | MRI artefact in the rectum caused by ingested orthodontic brackets. | 10.1016/j.radi.2017.10.002 | Almuqbel 2018 |
| 53 | Late-onset hydrocephalus in a child with Joubert syndrome: a case report. | 10.1007/s00381-018-3767-0 | Fehrenbach 2018 |
| 54 | Reduced corticostriatal functional connectivity in temporomandibular disorders. | 10.1002/hbm.24023 | He 2018 |
| 55 | Hypoglossal nerve paralysis in a child after a dental procedure. | 10.1016/j.pjnns.2018.01.006 | Marino 2018 |
| 56 | Delayed and incomplete treatment may result in dural fistula development in children with Vein of Galen malformation. | 10.1177/1591019917741755 | Meila 2018 |
| 57 | A Common Ancestral Asn242Ser Mutation in TMEM67 Identified in Multiple Iranian Families with Joubert Syndrome. | 10.1159/000477560 | Dehghani 2017 |
| 58 | Joubert Syndrome. | PMID: 20301500 | Parisi 1993 |
| 59 | Adaptive change in chewing-related brain activity while wearing a palatal plate: an functional magnetic resonance imaging study. | 10.1111/joor.12541 | Inamochi 2017 |
| 60 | Mutations in ARMC9, which Encodes a Basal Body Protein, Cause Joubert Syndrome in Humans and Ciliopathy Phenotypes in Zebrafish. | 10.1016/j.ajhg.2017.05.010 | VanDeWeghe 2017 |
| 61 | Hypomorphic mutations in POLR3A are a frequent cause of sporadic and recessive spastic ataxia. | 10.1093/brain/awx095 | Minnerop 2017 |
| 62 | The effects of a common stainless steel orthodontic bracket on the diagnostic quality of cranial and cervical 3T- MR images: a prospective, case-control study. | 10.1259/dmfr.20170051 | Cassetta 2017 |
| 63 | The usefulness of diagnostic imaging for the assessment of pain symptoms in temporomandibular disorders. | 10.1016/j.jdsr.2016.04.004 | Suenaga 2016 |
| 64 | Orocraniofacial findings of a Pediatric Patient with Joubert Syndrome. | 10.5005/jp-journals-10005-1394 | Goswami 2016 |
| 65 | A neonate with Joubert syndrome presenting with symptoms of Horner syndrome. | 10.3345/kjp.2016.59.11.S32 | Lee 2016 |
| 66 | Comparison of 2D single-shot turbo-spin-echo and spin-echo echo-planar diffusion weighted brain MRI at 3.0 Tesla: preliminary experience in children. | 10.1016/j.clinimag.2016.12.005 | Pokorney 2017 |
| 67 | Functional brain mapping using specific sensory-circuit stimulation and a theoretical graph network analysis in mice with neuropathic allodynia. | 10.1038/srep37802 | Komaki 2016 |
| 68 | Increased bone turnover, osteoporosis, progressive tibial bowing, fractures, and scoliosis in a patient with a final-exon SATB2 frameshift mutation. | 10.1002/ajmg.a.37847 | Boone 2016 |
| 69 | Biallelic Mutations of VAC14 in Pediatric-Onset Neurological Disease. | 10.1016/j.ajhg.2016.05.008 | Lenk 2016 |
| 70 | Altered neural activation pattern during teeth clenching in temporomandibular disorders. | 10.1111/odi.12465 | He 2016 |
| 71 | Nystagmus in a newborn: a manifestation of Joubert syndrome in the neonatal period. | 10.1136/bcr-2015-213127 | Salva 2016 |
| 72 | An unusual presentation of Joubert syndrome and related disorders in a newborn: Panhypopituitarism |  | Erol 2016 |
| 73 | Induced magnetic moment in stainless steel components of orthodontic appliances in 1.5 T MRI scanners. | 10.1118/1.4930796 | Wang 2015 |
| 74 | [Clinical and genetic analysis for a Joubert syndrome family with CC2D2A gene mutations]. |  | Su 2015 |
| 75 | Uncrossed epileptic seizures in Joubert syndrome. | 10.1136/bcr-2014-207719 | LÃ³pezRuiz 2015 |
| 76 | To see bruxism: a functional MRI study. | 10.1259/dmfr.20150019 | YÄ±lmaz 2015 |
| 77 | MRI with intraoral orthodontic appliance-a comparative in vitro and in vivo study of image artefacts at 1.5â€‰T. | 10.1259/dmfr.20140416 | Zachriat 2015 |
| 78 | Impact of orthodontic appliances on the quality of craniofacial anatomical magnetic resonance imaging and real-time speech imaging. | 10.1093/ejo/cju103 | Wylezinska 2015 |
| 79 | Occluso-facial manifestations in a child with the Rasmussen syndrome and intercurrent Lyme disease--a case report. | PMID: 26982755 | Cudzilo 2015 |
| 80 | The Shepherd's Crook Sign: A New Neuroimaging Pareidolia in Joubert Syndrome. | 10.1111/jon.12159 | Manley 2015 |
| 81 | Spontaneous neural activity alterations in temporomandibular disorders: a cross-sectional and longitudinal resting-state functional magnetic resonance imaging study. | 10.1016/j.neuroscience.2014.07.067 | He 2014 |
| 82 | Anesthetic management of patients with Joubert syndrome: a retrospective analysis of a single-institutional case series. | 10.1111/pan.12472 | Sriganesh 2014 |
| 83 | The time-course change of brain activity during learning new movement patterns of pronunciation with orthodontic appliances and its correlation with self-evaluation of pronunciation difficulty: an fMRI study | PMID: 24812764 | Kaneshima 2014 |
| 84 | Assessing the MR compatibility of dental retainer wires at 7 Tesla. | 10.1002/mrm.25019 | Wezel 2014 |
| 85 | Effects of cortical activations on enhancement of handgrip force during teeth clenching: an fMRI study. | 10.1016/j.neures.2013.11.006 | Kawakubo 2014 |
| 86 | Fetal eye movements on magnetic resonance imaging. | 10.1371/journal.pone.0077439 | Woitek 2013 |
| 87 | Goldenhar syndrome and medulloblastoma: a coincidental association? The first case report. | 10.1016/j.jcms.2013.07.003 | Aizenbud 2014 |
| 88 | Refined methodology for implantation of a head fixation device and chronic recording chambers in non-human primates. | 10.1016/j.jneumeth.2013.07.015 | Lanz 2013 |
| 89 | Joubert syndrome: the molar tooth sign of the mid-brain. | 10.4103/2141-9248.113686 | Nag 2013 |
| 90 | Clinical presentation of epignathus teratoma with cleft palate; and duplication of cranial base, tongue, mandible, and pituitary gland. | 10.1097/SCS.0b013e3182953b1f | Maeda 2013 |
| 91 | Joubert syndrome and related disorders. | 10.1016/B978-0-444-  59565-2.00058-7 | Valente 2013 |
| 92 | The diagnostic utility of exome sequencing in Joubert syndrome and related disorders. | 10.1038/jhg.2012.117 | Tsurusaki 2013 |
| 93 | Feasibility of clinical magnetoencephalography (MEG) functional mapping in the presence of dental artefacts. | 10.1016/j.clinph.2012.06.013 | Hillebrand 2013 |
| 94 | DÃ©jÃ  vu all over again: continuous treatment and the statute of limitations. | 10.1016/j.ajodo.2012.03.016 | Jerrold 2012 |
| 95 | Genotype-phenotype correlation in CC2D2A-related Joubert syndrome reveals an association with ventriculomegaly and seizures. | 10.1136/jmedgenet-2011-100552 | Bachmann-Gagescu 2012 |
| 96 | Joubert syndrome and related disorders, prenatal diagnosis with ultrasound and magnetic resonance imaging. | 10.5152/jtgga.2011.75 | Iskender 2012 |
| 97 | Perspectives. | 10.3174/ajnr.A2929 | Castillo 2012 |
| 98 | Self-face evaluation and self-esteem in young females: an fMRI study using contrast effect. | 10.1016/j.neuroimage.2011.10.098 | Oikawa 2012 |
| 99 | A neonate with developmental retardation | PMID: 21382210 | Bloemenkamp 2011 |
| 100 | Cockayne syndrome | PMID: 21342626 | Wang 2011 |
| 101 | Motor control of jaw movements: An fMRI study of parafunctional clench and grind behavior. | 10.1016/j.brainres.2011.01.096 | Wong 2011 |
| 102 | Orbital apex syndrome after tooth extraction in an immunocompromised patient. | 10.3980/j.issn.2222-3959.2011.01.26 | Subramaniam 2011 |
| 103 | Brain malformation in single median maxillary central incisor. | 10.1055/s-0030-1248245 | Kjaer 2009 |
| 104 | Clinical and molecular features of Joubert syndrome and related disorders. | 10.1002/ajmg.c.30229 | Parisi 2009 |
| 105 | Eye movement abnormalities in Joubert syndrome. | 10.1167/iovs.08-3299 | Weiss 2009 |
| 106 | Joubert syndrome associated with new MRI findings and posterior reversible encephalopathy syndrome. | PMID: 19402575 | Yerdelen 2009 |
| 107 | fMRI study of brain activity elicited by oral parafunctional movements. | 10.1111/j.  1365-2842.2009.01947.x | Byrd 2009 |
| 108 | Artifacts in brain magnetic resonance imaging due to metallic dental objects. | PMID: 19300375 | Costa 2009 |
| 109 | The neurobiology of swallowing and dysphagia. | 10.1002/ddrr.12 | Miller 2008 |
| 110 | The neuroanatomic basis of facial perception and variable facial discrimination ability: implications for orthodontics. | 10.1016/j.ajodo.2006.04.031 | Masella 2007 |
| 111 | Functional magnetic resonance imaging of brain activity during chewing and occlusion by natural teeth and occlusal splints. | 10.1016/j.aanat.2007.02.027 | Kordass 2007 |
| 112 | Meckel syndrome in the Hutterite population is actually a Joubert-related cerebello-oculo-renal syndrome. | 10.1002/ajmg.a.31832 | Boycott 2007 |
| 113 | Joubert syndrome with atrial septal defect and persistent left superior vena cava. | PMID: 17562515 | Elmali 2007 |
| 114 | Joubert syndrome (and related disorders) (OMIM 213300). | 10.1038/sj.ejhg.5201648 | Parisi 2007 |
| 115 | Lack of NPHP2 mutations in a newborn infant with Joubert syndrome-related disorder presenting as end-stage renal disease. | 10.1007/s00467-006-0412-z | Assadi 2007 |
| 116 | MRI scanning and orthodontics. | 10.1179/146531205225021726 | Patel 2006 |
| 117 | Bilateral congenital trigeminal sensorimotor neuropathy presents as a severe open-bite malocclusion. | 10.1097/01.scs.  0000221519.65114.84 | GÃ¼zel 2006 |
| 118 | Effects of mandibular advancement on brain activation during inspiratory loading in healthy subjects: a functional magnetic resonance imaging study. | 10.1152/japplphysiol.00169.  2005 | Hashimoto 2006 |
| 119 | AHI1 mutations cause both retinal dystrophy and renal cystic disease in Joubert syndrome. | 10.1136/jmg.2005.036608 | Parisi 2006 |
| 120 | Head motion analysis during cognitive fMRI examination: application in patients with schizophrenia. | 10.1016/j.neures.2005.06.004 | Yoo 2005 |
| 121 | Elevated levels of beta-endorphin in temporomandibular joint synovial lavage fluid of patients with closed lock. | PMID: 15779538 | Kajii 2005 |
| 122 | De novo paracentric inversion 14q13q24.1 in a patient with severe involuntary movements, epilepsy, oligodontia and dysmorphic features. | PMID: 15517827 | Peippo 2004 |
| 123 | Joubert syndrome: long-term follow-up. | 10.1017/s0012162204001161 | Hodgkins 2004 |
| 124 | The NPHP1 gene deletion associated with juvenile nephronophthisis is present in a subset of individuals with Joubert syndrome. | 10.1086/421846 | Parisi 2004 |
| 125 | The human parietal cortex is involved in spatial processing of tongue movement-an fMRI study. | 10.1016/j.neuroimage.2003.  10.024 | Watanabe 2004 |
| 126 | Life-threatening respiratory failure due to cranial dystonia after dental procedure in a patient with multiple system atrophy. | 10.1002/mds.10456 | Hamzei 2003 |
| 127 | Report of a patient with hypoglossia-hypodactylia syndrome and a review of the literature. | 10.1597/1545-1569_2003  _040_0196_roapwh_2.0.co_2 | Yasuda 2003 |
| 128 | Three-dimensional magnetic resonance image of the mandible and masticatory muscles in a case of juvenile chronic arthritis treated with the Herbst appliance. | 10.1043/0003-3219(2002)072  <0081:TDMRIO>2.0.CO;2 | Kitai 2002 |
| 129 | A case of idiopathic brain calcification associated with dyschromatosis symmetrica hereditaria, aplasia of dental root, and aortic valve sclerosis | PMID: 11771159 | Tojyo 2001 |
| 130 | Joubert's syndrome: report of 12 cases | PMID: 11424029 | Barreirinho 2001 |
| 131 | Clinical nosologic and genetic aspects of Joubert and related syndromes. | 10.1177/088307389901401007 | Chance 1999 |
| 132 | Joubert syndrome: monozygotic twins with discordant phenotypes. | 10.1177/088307389901401005 | Raynes 1999 |
| 133 | Median facial dysplasia in patients with lip-jaw-palate clefts. Characteristics and problems in interdisciplinary orthodontic-oral surgery treatment | 10.1007/BF03043537 | Opitz 1997 |
| 134 | A case of hereditary motor and sensory neuropathy with vocal cords palsy and diaphragmatic weakness | PMID: 8319389 | Fukuda 1993 |
| 135 | Permanent bruxism as a manifestation of the oculo-facial syndrome related to systemic Whipple's disease. | 10.1002/mds.870070117 | Tison 1992 |
| 136 | Dental material artifacts on MR images. | 10.1148/radiology.166.3.  3340777 | HinshawDBJr 1988 |
| 137 | Orthodontic appliances and magnetic resonance imaging of the brain and temporomandibular joint. | 10.1043/0003-3219(1988)058  <0009:OAAMRI>2.0.CO;2 | Sadowsky 1988 |
| 138 | High-field MRI and Mercury release from dental amalgam fillings |  | Mortazavi 2014 |
| 139 | An Internet-based Multimodal Pain Program for Chronic Temporomandibular Disorder Pain | PMID: 33048053 | NCT04363762 2020 |
| 140 | Positive Aging: the Impact of a Community Wellbeing and Resilience Program | 10.1080/07317115.2018.  1561582 | Bartholomaeus 2019 |
| 141 | Effects of Physical Activity Intervention for Chinese People With Severe Mental Illness | 10.1177/1049731518804900 | Young 2019 |
| 142 | Psychometric properties of the Child Oralâ€care Performance Assessment Scale | 10.1111/cdoe.12476 | Nair 2019 |
| 143 | Online Simulation-Based Mastery Learning with Deliberate Practice: developing Interprofessional Communication Skill | 10.1016/j.ecns.2019.04.005 | Yeh 2019 |
| 144 | The development and use of the assessment of dementia awareness and person-centred care training tool in long-term care | 10.1177/1471301218768165 | Creese 2019 |
| 145 | Cognitive Changes and Neural Correlates After Rehabilitation of Masticatory Function in Elderly -an Intervention Study |  | NCT04458207 2020 |
| 146 | Citicoline for treating people with acute ischemic stroke | 10.1002/14651858.CD013066.pub2 | MartÃ­-Carvajal 2020 |
| 147 | Speech therapy for children with dysarthria acquired before three years of age | 10.1002/14651858.  CD006937.pub3 | Pennington 2016 |
| 148 | Methylphenidate for attention deficit hyperactivity disorder (ADHD) in children and adolescents â€“ assessment of adverse events in nonâ€randomised studies | 10.1002/14651858.  CD012069.pub2 | StorebÃ¸ 2018 |
| 149 | Exercise interventions for cerebral palsy | 10.1002/14651858.  CD011660.pub2 | Ryan 2017 |
| 150 | Radiological assessment of facial recess and correlation with surgical measurement in cochlear implantation | 10.1186/s43163-023-00438-8 | Behairy 2023 |
| 151 | Hakuba-Dolenc Approach for Resection of a Large Cavernous Sinus Dermoid Cyst: 2-Dimensional Operative Video | 10.1227/ons.  0000000000000733 | Sun 2023 |
| 152 | SALL4 phenotype in four generations of one family: An interplay of the upper limb, kidneys, and the pituitary | 10.1159/000531996 | KodytkovÃ¡ 2023 |
| 153 | Clinical and therapeutic course in head variants of linear morphea in adults: a retrospective review | 10.1007/s00403-022-02478-1 | Fan 2023 |
| 154 | Saw-tooth vertical saccades in progressive supranuclear palsy | 10.1007/s00415-023-11696-0 | Kim 2023 |
| 155 | Causal Interactions in Brain Networks Predict Pain Levels in Trigeminal Neuralgia | 10.1101/2023.06.02.23290885 | Liang 2023 |
| 156 | Joubert Syndrome with Renal and Cerebral Manifestations: A Case Series of Three Siblings | 10.7860/JCDR  /2023/63675.18046 | Kumar 2023 |
| 157 | A mutational hotspot in AMOTL1 defines a new syndrome of orofacial clefting, cardiac anomalies, and tall stature | 10.1002/ajmg.a.63130 | Strong 2023 |
| 158 | SOX5: Lambâ€“Shaffer syndromeâ€”A case series further expanding the phenotypic spectrum | 10.1002/ajmg.a.63124 | Edgerley 2023 |
| 159 | A Real Pain in the Neck | 10.1097/  JSM.0000000000001125 | Castro 2023 |
| 160 | Identification and classification of a childhood form of hereditary hypophosphatasia | 10.1038/s41431-023-01339-3 | Szegedi 2023 |
| 161 | Joubert Syndrome: A Rare Cause of Progressive Chronic Respiratory Failure | 10.1164/ajrccm-  conference.2023.A55 | Kharode 2023 |
| 162 | Pre-Epiglottic Baton Plate in the Management of Upper Airway Obstruction in an Infant with Femoral Facial Syndrome: A Case Report | 10.1177/10556656211059705 | Pang 2023 |
| 163 | Expanding the neurodevelopmental phenotype associated with HK1 de novo heterozygous missense variants | 10.1016/j.ejmg.2023.104696 | Poole 2023 |
| 164 | Expanding the CANVAS: A rare case from South East Asia | 10.1002/mdc3.13679 | KoyaKutty 2023 |
| 165 | A curious case of 25 digit polysyndactyly and congenital laryngeal anomalies | 10.1016/S0002-9629  (23)00341-5 | Carey 2023 |
| 166 | Seizure Caused by Intraparenchymal Hemorrhage from Migration of Mandibular Dental Wire through Foramen Ovale in a Child: A Case Report | 10.1055/s-0043-1762237 | Morgan 2023 |
| 167 | Japanese Orthopaedic Association (JOA) Clinical practice guidelines on the Management of Cervical Spondylotic Myelopathy,2020 â€“ Secondary publication | 10.1016/j.jos.2022.03.012 | Watanabe 2023 |
| 168 | Expanding the spectrum of KIF5A mutationsâ€”case report of a large kindred with familial ALS and overlapping syndrome | 10.1080/21678421.2022.2164204 | Dulski 2023 |
| 169 | Locus Coeruleus Dysfunction and Trigeminal Mesencephalic Nucleus Degeneration: A Cue for Periodontal Infection Mediated Damage in Alzheimerâ€™s Disease? | 10.3390/ijerph20021007 | Pisani 2023 |
| 170 | Single Bundle A.C.L. Reconstruction in Partial Injuries of A.C.L | 10.47750/pnr.2023.14.S02.106 | ElTohamy 2023 |
| 171 | Pathological Changes of Small Vessel Disease in Intracerebral Hemorrhage: a Systematic Review and Meta-analysis | 10.1007/s12975-023-01154-4 | Xu 2023 |
| 172 | Adult Vanishing White Matter Disease with a Novel EIF2B4 Mutation | 10.29271/jcpsp.2022.  JCPSPCR.CR181 | Su 2022 |
| 173 | A NOVELMUTATION IN COA7 LEADING TO FRIEDRICH'S LIKE ATAXIA | 10.1007/s10072-022-06531-9 | Setola 2022 |
| 174 | Optic pathway tumor in an infant with KIAA1109 variants | 10.1002/pbc.29701 | Shah 2022 |
| 175 | Cerebrospinal fluid biomarkers of white matter injury and astrogliosis are associated with the severity and surgical outcome of degenerative cervical spondylotic myelopathy | 10.1016/j.spinee.2022.06.012 | Tsitsopoulos 2022 |
| 176 | Somatosensory evoked magnetic fields induced by electrical palate stimulation in patients with unilateral cleft lip and palate after palatoplasty | 10.1016/j.neures.2022.08.002 | Shimada 2022 |
| 177 | Mitochondrial respiratory chain dysfunction in a patient with a heterozygous de novo CTBP1 variant | 10.1002/jmd2.12326 | Wong 2022 |
| 178 | Factors Affecting Slip Reduction in Oblique Lumbar Interbody Fusion With Posterior Fixation for Degenerative Spondylolisthesis | 10.1177/2192568221989295 | Chung 2022 |
| 179 | Is bruxism associated with changes in neural pathways? A systematic review and meta-analysis of clinical studies using neurophysiological techniques | 10.1007/s11682-021-00601-w | Boscato 2022 |
| 180 | Biallelic variants in WARS1 cause a highly variable neurodevelopmental syndrome and implicate a critical exon for normal auditory function | 10.1002/humu.24435 | Lin 2022 |
| 181 | A CLOT OR NOT A CLOT: THAT IS THE QUESTION | 10.1016/j.chest.2022.08.599 | Martinez 2022 |
| 182 | Multiple brown tumors within the cranium, in a patient with primary hyperparathyroidism. A case report and review of the literature | 10.1016/j.inat.2022.101592 | Bardeesi 2022 |
| 183 | TRPV4 Mutation related Parkinson's Disease with Scapuloperoneal spinal muscular atrophy | 10.1002/mds.29223 | Deenadayalu 2022 |
| 184 | DBS Emergency Surgery for Treatment of Dystonic Storm Associated with Rhabdomyolysis and Acute Colitis in GNAO1 Mutation | 10.1159/000526735 | Chaib 2022 |
| 185 | Stroke incidence and chronic kidney disease: A hospital-based prospective cohort study | 10.1111/nep.14049 | Wakasugi 2022 |
| 186 | Paroxysmal dyskinesia in a case of hypocalcemia with hypothyroidism and pseudohypoparathyroidism | 10.4103/aian.aian-896-21 | Niveditha 2022 |
| 187 | S100-negative epithelioid malignant peripheral nerve sheath tumor with possible perineurial differentiation | 10.1007/s00428-021-03218-y | Yamashita 2022 |
| 188 | SYDENHAM'S CHOREA IN AN ELDERLY PATIENT | 10.1136/jnnp-2022-ABN.259 | Diyab 2022 |
| 189 | A Scoping Review of Injuries in Amateur and Professional Menâ€™s Ice Hockey | 10.1177/23259671221085968 | Szukics 2022 |
| 190 | Alteration of Oral and Perioral Soft Tissue in Mice following Incisor Tooth Extraction | 10.3390/ijms23062987 | Takagi 2022 |
| 191 | eP203: Genotype/phenotype correlations in Joubert syndrome | 10.1016/j.gim.2022.01.239 | Owens 2022 |
| 192 | Orthopaedic Aspects of SAMS Syndrome | 10.1055/s-0040-1714700 | Schrander 2022 |
| 193 | Transient Monocular Visual Loss Induced By Celecoxib: A Rare Adverse Effect | 10.53350/pjmhs22162926 | Al-Harbi 2022 |
| 194 | Two Siblings Showing a Mild Phenotype of Joubert Syndrome with a Specific CEP290 Variant | 10.1055/a-1865-6890 | Uda 2022 |
| 195 | Corticocortical paired associative stimulation for treating motor dysfunction after stroke: Study protocol for a randomised sham-controlled double-blind clinical trial | 10.1136/bmjopen-2021-053991 | Duan 2022 |
| 196 | The global burden of adolescent and young adult cancer in 2019: a systematic analysis for the Global Burden of Disease Study 2019 | 10.1016/S1470-  2045(21)00581-7 | Alvarez 2022 |
| 197 | Dissecting aneurysm of the anterior inferior cerebellar artery in the internal auditory canal presenting with deafness without hemorrhage: A case report and literature review | 10.25259/SNI_1220_2021 | Okada 2022 |
| 198 | Adjacent Segment Stenosis after Muscle-Preserving Selective Laminectomy: A Retrospective Study of Patients with a Minimum 10-Year Follow-Up | 10.22603/ssrr.2021-0076 | Aoyama 2022 |
| 199 | Whole exome sequencing facilitated the diagnosis in four Chinese pediatric cases of Joubert syndrome related disorders | PMID: 35958498 | Zhang 2022 |
| 200 | LAMA1 variants were identified Joubert syndrome patient | 10.1111/ped.14980 | Noguchi 2022 |
| 201 | Artifacts Of MRI In Oromaxillofacial Region-Systematic Review And Meta Analysis | 10.47750/pnr.2022.13.S10.300 | UdayiniMonica 2022 |
| 202 | An Atypical Case of Optic Neuropathy | 10.1001/  jamaophthalmol.2021.1466 | Williams 2022 |
| 203 | Reducing the burden of care: Multidisciplinary management of late-manifested crouzon syndromeâ€”a case report | 10.3390/children8121122 | Achterrath 2021 |
| 204 | Evaluation of Medial Meniscal Thickness, Tear and Extrusion in Patients with and without Osteoarthritis of Knee: A Cross-sectional Study | 10.7860/JCDR/2021/50637.15752 | Singh 2021 |
| 205 | Mitofusin 2: The missing link between mtDNA maintenance defects and neurotransmitter disorders | 10.1016/j.mito.2021.09.011 | Pellino 2021 |
| 206 | Management of severely aberrant permanent first molars in molar rootâ€“incisor malformation patients: Case series and a guideline | 10.3390/children8100904 | Song 2021 |
| 207 | ACUTE ACQUIRED MACROGLOSSIA IN REFRACTORY STATUS EPILEPTICUS PATIENT | 10.1016/j.chest.2021.07.709 | MaaliMohamed 2021 |
| 208 | Management of tremor secondary to MORC2 related neurodevelopmental disorder with deep brain stimulation | 10.1002/mds.28794 | Hull 2021 |
| 209 | Mirror Movements of the Left Hand in a Patient with Joubert Syndrome | 10.1002/mdc3.13256 | Ismail 2021 |
| 210 | Serum C-reactive protein in dogs with paraplegia secondary to acute intervertebral disc extrusion | 10.1111/jvim.16179 | Foreman 2021 |
| 211 | Management of apical periodontitis using WaveOne gold reciprocating files, single-cone endodontic approach: A case series author | 10.1016/j.amsu.2021.102385 | MahmoodTalabani 2021 |
| 212 | Case report-a case of rothia denticariosa endocarditis with severe complications | 10.1136/jim-2021-MW.56 | Bak 2021 |
| 213 | Evaluation of oral hygiene status in patients with hemorrhagic and ischemic stroke | 10.4103/jpbs.JPBS_698_20 | Sinha 2021 |
| 214 | Diagnosis, early care, and treatment of hallermann-streiff syndrome: A review of the literature | 10.3928/19382359-  20210415-01 | Godzieba 2021 |
| 215 | Cleft lip in oto-palato-digital syndrome type I | 10.1111/cga.12409 | Kusajima 2021 |
| 216 | A case report of Joubert syndrome with renal involvement and seizures in a neonate | 10.1016/j.radcr.2021.02.031 | Ahmetgjekaj 2021 |
| 217 | Randomized double-blind sham-controlled trial of thalamic versus GPi stimulation in patients with severe medically refractory Gilles de la Tourette syndrome | 10.1016/j.brs.2021.04.004 | MÃ¼ller-Vahl 2021 |
| 218 | Dental and Craniofacial Characteristics in Patients With 14Q22.1-Q22.2 Deletion: A Case Series | 10.1177/1055665620954090 | Roelandt 2021 |
| 219 | Aberrant splicing in gjb1 and the relevance of 5â€² utr in cmtx1 pathogenesis | 10.3390/brainsci11010024 | Boso 2021 |
| 220 | Lumbar interlaminar epidural steroid injection - Experience at Mayo Hospital Lahore |  | Hussain 2021 |
| 221 | Joubert syndrome associated with central sleep apnea in an adult from Colombia | 10.46997/  REVECUATNEUROL30100206 | Quintero-Moreno 2021 |
| 222 | POLR3A-related hypomyelinating leukodystrophy: case report and literature review | 10.17650/2222-8721-  2021-11-4-48-54 | Murtazina 2021 |
| 223 | Imaging Approach to Conductive Hearing Loss | 10.1007/978-3-030-70635-7_3 | Pulickal 2021 |
| 224 | Severe unremitting headache unresponsive to indomethacin after receiving second-dose of COVID-19 mrna vaccine in patient with hemicrania continua: A case report | 10.1002/pmrj.12735 | Yazdanpanah 2021 |
| 225 | Facial fractures: classification and highlights for a useful report | 10.1186/s13244-020-00847-w | GÃ³mezRosellÃ³ 2020 |
| 226 | Arthroscopic diagnosis and medical management of calcium pyrophosphate deposition disease in the temporomandibular joint | 10.1016/j.ijom.2020.07.029 | Choi 2020 |
| 227 | CMT4J, parkinsonism and a new FIG4 mutation | 10.1016/j.parkreldis.2020.10.011 | Posada 2020 |
| 228 | Analysis of the impact of spinopelvic radiographic parameters on the severity of cervical spondylotic myelopathy | 10.1016/j.jos.2020.01.006 | Ninomiya 2020 |
| 229 | Choreoacanthocytosis in a Sri Lankan family presenting with 70% of acanthocytes in a peripheral blood film | 10.1016/j.parkreldis.  2020.06.399 | Sudath 2020 |
| 230 | Plantar stimulation alters brain connectivity in idiopathic Parkinson's disease | 10.1111/ane.13253 | Pagnussat 2020 |
| 231 | Effectiveness of a sensor-based technology in upper limb motor recovery in post-acute stroke neurorehabilitation: A randomized controlled trial |  | Tramontano 2020 |
| 232 | Lateral cephalogram and CBCT as a diagnostic aid for analysis of airway- Review article |  | Banerjee 2020 |
| 233 | POLR3A Leukodystrophy presenting with levodopa responsive parkinsonism | 10.1002/mds.28268 | Kyle 2020 |
| 234 | KMT2B-related dystonia (DYT28) and Klippel-Feil Syndrome: A new association? | 10.1002/mds.28268 | Portela 2020 |
| 235 | A case of advanced ankylosing spondylitis complicated with cervical myelopathy due to ossification of yellow ligament in which bone histomorphometry demonstrated delayed calcification | 10.1080/24725625.  2019.1702496 | Kondo 2020 |
| 236 | Sex-Related Differences in the Effects of Sports-Related Concussion: A Review | 10.1111/jon.12726 | Koerte 2020 |
| 237 | Oral-Facial-Digital Syndrome Type 1: Further Clinical and Molecular Delineation in 2 New Families | 10.1177/1055665620902880 | Faily 2020 |
| 238 | Rare cause of Hypomyelinating Leukodystrophy type 7: â€œGly672Glu homozygous variant of the POLR3A geneâ |  | CarvalhoMonteiro 2020 |
| 239 | Short-term effects of flupentixol-melitracen on regional brain function in patients with irritable bowel syndrome: An fmri study | 10.5812/iranjradiol.91835 | Li 2020 |
| 240 | Functional appliances in the treatment and management of mandibular retrognathism-a literature review | 10.31838/ijpr/2020.12.02.375 | Nishanthi 2020 |
| 241 | Comparison between muscle-preserving selective laminectomy and laminoplasty for multilevel cervical spondylotic myelopathy | 10.21037/atm.2019.11.132 | Nori 2020 |
| 242 | Context-guided fully convolutional networks for joint craniomaxillofacial bone segmentation and landmark digitization | 10.1016/j.media.2019.101621 | Zhang 2020 |
| 243 | A comprehensive review of adult onset spinal teratomas: analysis of factors related to outcomes and recurrences | 10.1007/s00586-019-06037-7 | Prasad 2020 |
| 244 | Outcomes of Surgery for Thoracic Myelopathy Owing to Thoracic Ossification of the Ligamentum Flavum in a Nationwide Multicenter Prospectively Collected Study in 223 Patients: Is Instrumented Fusion Necessary? | 10.1097/BRS.  0000000000003208 | Ando 2020 |
| 245 | Adult-onset POLR3 leukodystrophy: A case report | 10.1002/mdc3.12905 | Patel 2020 |
| 246 | Translational research of temporomandibular joint pathology: A preliminary biomarker and fMRI study | 10.1186/s12967-019-02202-0 | Barkhordarian 2020 |
| 247 | Craniofacial asymmetry from one to three years of age: A prospective cohort study with 3D imaging | 10.3390/jcm9010070 | Launonen 2020 |
| 248 | Extraordinary case presentations in pediatric pituitary adenoma: Report of 6 cases | 10.3171/2019.7.PEDS1950 | Meyer 2020 |
| 249 | Unusual neuromuscular presentation of a Wilsonâ€™s disease patient with one-stage surgical correction treatment: A case report | 10.1177/2309499020934053 | Lin 2020 |
| 250 | An adolescent with herpes simplex encephalitis, presenting with mild symptoms and rapid deterioration: A case report | 10.1177/2050313X20977142 | Stepien 2020 |
| 251 | Multiple sclerosis diagnosed in patients presenting with trigeminal neuralgia at oral medicine department, khyber college of dentistry, peshawar |  | Khan 2020 |
| 252 | Joubert's syndrome with dystonic-ataxic tremor: A novel phenotypic variant |  | Larson 2020 |
| 253 | Neurodevelopmental regression, severe generalized dystonia, and metabolic acidosis caused by POLR3A mutations | 10.1212/NXG.  0000000000000521 | Zanette 2020 |
| 254 | Multimodal rehabilitation for Foix-Chavany-Marie syndrome (FCMS): A case report | 10.1002/pmrj.12271 | Qiu 2019 |
| 255 | Muenke syndrome: Long-term outcome of a syndrome-specific treatment protocol | 10.3171/2019.5.PEDS1969 | denOttelander 2019 |
| 256 | Thoracic myelopathy caused by an extremely rare aberrant epidural ligament: A case report | 10.1097/MD.  0000000000017344 | Hirai 2019 |
| 257 | Orthostatic jaw tremor, a diagnostic challenge | 10.11648/j.cnn.20200403.14 | Moraes 2019 |
| 258 | Hypophosphatasia presenting as parkinsonism with compound heterozygous mutations in ALPL gene |  | Yu 2019 |
| 259 | Assessment of articular disc position before and after gnathological splint therapy in symptomatic temporomandibular disorder patients using magnetic resonance imaging | 10.5958/0976-5506.  2019.02360.X | Robin 2019 |
| 260 | Cranioplasty: A new perspective | 10.3889/oamjms.2019.489 | MostafaElkatatny 2019 |
| 261 | The cerebellar phenotype of Charcot-Marie-Tooth neuropathy type 4C | 10.1186/s40673-019-0103-8 | Skott 2019 |
| 262 | The influence of suturectomy on age-related changes in cerebral blood flow in rabbits with familial bicoronal suture craniosynostosis: A quantitative analysis | 10.1371/journal.pone.  0197296 | Grandhi 2019 |
| 263 | In-depth characterization of congenital Zika syndrome in immunocompetent mice: Antibody-dependent enhancement and an antiviral peptide therapy | 10.1016/j.ebiom.2019.05.014 | Camargos 2019 |
| 264 | A case of functional dystonia: clinical cues to differentiate between organic and functional dystonia | 10.1007/s10072-018-3654-1 | Demartini 2019 |
| 265 | Cerebral hypomyelination associated with biallelic variants of FIG4 | 10.1002/humu.23720 | Lenk 2019 |
| 266 | Compartment syndrome of the muscles of mastication in a working dog following a traumatic training incident | 10.1111/vec.12829 | Brida 2019 |
| 267 | Comparison of 2D turbo-spin-echo blade and spin-echo echo-planar diffusion weighted brain MRI at 3 tesla: Preliminary experience in children | 10.1007/s00247-019-04376-7 | McAllister 2019 |
| 268 | A case of unremitting ear pain | 10.1111/jgs.15898 | Hamilton 2019 |
| 269 | Rare presentations of cranial nerve dysfunction associated with space-occupying lesions | 10.1111/jop.12796 | Amanda 2019 |
| 270 | Clinical report of 8 patients with 49,XXXXY syndrome: Delineation of the facial gestalt and depiction of the clinical spectrum | 10.1016/j.ejmg.2018.07.016 | Burgemeister 2019 |
| 271 | Inclination of the condylar long axis is not related to temporomandibular disc displacement | 10.1111/jicd.12375 | Amorim 2019 |
| 272 | Sturge-weber syndrome: A case report with brief review of literature |  | Patil 2019 |
| 273 | Craniofacial soft and hard tissue symmetry depicted with MRI, based on integrated and modular organization of the human head: Three-dimensional analysis | ISSN:1940-5901/IJCEM0076660 | Jiang 2019 |
| 274 | Full-mouth rehabilitation of an acromegaly disease patient with removable prostheses: A clinical case report | 10.11604/pamj.  2019.33.5.18194 | Bekri 2019 |
| 275 | Prolonged Myelosuppression due to Progressive Bone Marrow Fibrosis in a Patient with Acute Promyelocytic Leukemia | 10.1155/2019/1616237 | Inagawa 2019 |
| 276 | Homozygous NPHP1 gene deletion of two patients with joubert syndrome | 10.1086/421846 | Kocagil 2019 |
| 277 | Image segmentation techniques for healthcare systems | 10.1155/2019/2723419 | Gambino 2019 |
| 278 | The frog Xenopus as a model to study Joubert syndrome: The case of a human patient with compound heterozygous variants in PIBF1 | 10.3389/fphys.2019.00134 | Ott 2019 |
| 279 | Is Modified K-line a Powerful Tool of Surgical Decision Making for Patients with Cervical Spondylotic Myelopathy? | 10.1097/BSD.  0000000000000899 | Hirai 2019 |
| 280 | Cerebral White Matter Lesions and Dysmorphisms: Signs Suggestive of 6p25 Deletion Syndrome-Literature Review | 10.1055/s-0039-1694015 | Pavone 2019 |
| 281 | Anti-NMDA receptor encephalitis presenting with status epilepticus: Brucellosis as a possible triggering factor: A case report | 10.4103/ijpvm.IJPVM-417-18 | Hakamifard 2019 |
| 282 | Three Patients with Joubert Syndrome and KIF7 gene mutations: Genotype phenotype correlation |  | Altundag 2019 |
| 283 | Sport-related concussion in children and adolescents | 10.1542/peds.2018-3074 | Halstead 2018 |
| 284 | Secondary dystonic tremor after medial medullary infarction |  | Wang 2018 |
| 285 | Task-specific embouchure dystonia in a flutist |  | Bledsoe 2018 |
| 286 | Severe leigh-like phenotype in an girl with homozygous P.Q139âˆ— mutation in C12ORF65 | 10.1055/s-0038-1675996 | Drenckhahn 2018 |
| 287 | Nontraumatic fracture: Do not miss seizure-Seizure: Do not miss nontraumatic fracture |  | Verma 2018 |
| 288 | Painful mydriasis as the only clinical sign of an ipsilateral midbrain abscess | 10.1016/j.clinph.2018.04.731 | ThÃ¶mke 2018 |
| 289 | Meta-analysis of brain mechanisms of chewing and clenching movements | 10.1111/joor.12657 | Lin 2018 |
| 290 | Lumbar epidural lipomatosis is associated with visceral fat and metabolic disorders | 10.1007/s00586-018-5584-2 | Morishita 2018 |
| 291 | Functional MRI and laser-evoked potentials evaluation in Charcot-Marie-Tooth syndrome | 10.1007/s10072-018-3401-7 | DeSalvo 2018 |
| 292 | Identification of CC2D1A homozygous mutation as a cause of Joubert Syndrome with obsessive compulsive disorder | 10.1002/2211-5463.12453 | Ergoren 2018 |
| 293 | De novo HDAC8 mutation causes Rett-related disorder with distinctive facial features and multiple congenital anomalies | 10.1016/j.braindev.  2017.12.013 | Saikusa 2018 |
| 294 | Medical applications of 3D printing |  | Jagadeesh 2018 |
| 295 | Severe macroglossia after posterior fossa and craniofacial surgery in children | 10.1016/j.ijom.2017.12.003 | Bouaoud 2018 |
| 296 | Intraorbital perineural cyst of the oculomotor nerve | CorpusID:59210294 | Mechtler 2018 |
| 297 | Congenital cavitary optic disc anomaly and Axenfeldâ€™s anomaly in Wolf-Hirschhorn syndrome: A case report and review of the literature | 10.1080/13816810.2017.  1408850 | Ali 2018 |
| 298 | Non-surgical transient cerebellar mutismâ€”case report and systematic review | 10.1007/s00381-017-3643-3 | Makarenko 2018 |
| 299 | Reach and grasp deficits following damage to the dorsal pulvinar | 10.1016/j.cortex.2017.10.011 | Wilke 2018 |
| 300 | Hypothalamic abnormalities: Growth failure due to defects of the GHRH receptor | 10.1016/j.ghir.2017.12.011 | Aguiar-Oliveira 2018 |
| 301 | Whole exome sequencing reveals novel compound heterozygous mutations in PIBF1 as a cause of Joubert syndrome | 10.1007/s11825-018-0176-4 | Evers 2018 |
| 302 | Orthodontist's view on cerebral palsy | 10.13005/bpj/1407 | Rajendran 2018 |
| 303 | The incidence and most common levels of thoracic degenerative disc pathologies | 10.5606/tftrd.2018.1302 | SarsÄ±lmaz 2018 |
| 304 | Autonomic and sensory neuropathy: Challenges in the etiology and treatment of a pediatric case | 10.3233/JND-189001 | Sampaio 2018 |
| 305 | Traumatic brain injury: Integrated approaches to improve prevention, clinical care, and research | 10.1016/S1474-  4422(17)30371-X | Maas 2017 |
| 306 | Parry Romberg Syndrom, uveitis and optic neuritis in a five year old child | 10.1016/j.jns.2017.08.546 | Hatteb 2017 |
| 307 | Evaluation of spinal conduction block in myelopaty patients by magnetospinography | 10.1016/j.jns.2017.08.2255 | Kawabata 2017 |
| 308 | Genetically proven marinesco sjogren syndrome from India: Rare combination of ataxia, myopathy and cataracts | 10.1016/j.jns.2017.08.2509 | Paidimarri 2017 |
| 309 | Case report: A rare cause of orofacial dyskinesia | 10.1016/j.jns.2017.08.2919 | Tan 2017 |
| 310 | Artifacts on temporomandibular joint MR images caused by mascara used as hair dye | 10.1007/s11282-016-0264-6 | Wakae-Morita 2017 |
| 311 | Anatomical Society Winter Meeting | 10.1111/joa.12637 | Burns 2017 |
| 312 | A new autosomal recessive amyelinating cause of Charcot Marie Tooth disease with CNS features and respiratory distress | 10.1111/jns.12225 | Siskind 2017 |
| 313 | Bardet-Biedl syndrome 3 regulates the development of cranial base midline structures | 10.1016/j.bone.2016.02.017 | Kawasaki 2017 |
| 314 | Goldenhar syndrome with rare clinical features | 10.1111/pde.13196 | Mittal 2017 |
| 315 | Mini titanium plates; hearkening the end of nonrigid cranial bone flap fixation | 10.12669/pjms.334.12003 | Junaid 2017 |
| 316 | A lateral cephalometry study of patients with neurofibromatosis type 1 | 10.1016/j.jcms.2017.02.011 | Friedrich 2017 |
| 317 | Hypomyelination with atrophy of the basal ganglia and cerebellum (H-ABC): A Mexican case report | 10.1002/mds.27087 | Kleinert-Altamirano 2017 |
| 318 | A case of GH deficiency in a patient with Joubert syndrome | 10.1136/archdischild-2017-313273.173 | Lanzano 2017 |
| 319 | Symmetry of fMRI activation in the primary sensorimotor cortex during unilateral chewing | 10.1007/s00784-016-1858-4 | Lotze 2017 |
| 320 | Loss-of-Function Mutations in LGI4, a Secreted Ligand Involved in Schwann Cell Myelination, Are Responsible for Arthrogryposis Multiplex Congenita | 10.1016/j.ajhg.2017.02.006 | Xue 2017 |
| 321 | Mutations in KIAA0753 cause Joubert syndrome associated with growth hormone deficiency | 10.1007/s00439-017-1765-z | Stephen 2017 |
| 322 | Moebius sequence -a multidisciplinary clinical approach | 10.1186/s13023-016-0559-z | Pedersen 2017 |
| 323 | Disruption of the Photoreceptor Inner Segmentâ€“Outer Segment Junction in a 6-Year-Old Girl with Joubert Syndrome | 10.1080/01658107.2016.1236391 | Baba 2017 |
| 324 | Novel CC2D2A compound heterozygous mutations cause Joubert syndrome | 10.3892/mmr.2016.6007 | Xiao 2017 |
| 325 | Experimental occlusal interference on brain activation during gum chewing |  | Otsuka 2017 |
| 326 | Correlation of clinical examination, MRI and arthroscopy findings in menisco-cruciate injuries of the knee: A prospective diagnostic study | 10.5812/atr.30364 | Panigrahi 2017 |
| 327 | Effects of Low-Frequency repetitive transcranial magnetic stimulation and neuromuscular electrical stimulation on upper extremity motor recovery in the early period after stroke: A preliminary study | 10.1080/10749357.2017.1305644 | Tosun 2017 |
| 328 | Actinomycosis presenting with septic cavernous sinus thrombosis | 10.1164/ajrccm-  conference.2017.A52 | BenFaras 2017 |
| 329 | Physiological effects of a habituation procedure for functional MRI in awake mice using a cryogenic radiofrequency probe | 10.1016/j.jneumeth.2016.09.013 | Yoshida 2016 |
| 330 | Joubert Syndrome Mimicking Hypotonic Cerebral Palsy | 10.1007/s12098-016-2196-x | kumar 2016 |
| 331 | A rare case of movement disorder - Cortico basalganglionic degeneration |  | Raj 2016 |
| 332 | Identification of a â€œCrypticâ€ de novo deletion in NKX2.1 in the Brain-Lung-Thyroid syndrome using genomic SNP arrays | 10.1159/000449142 | Villafuerte 2016 |
| 333 | A severe spinal muscular atrophy phenotype associated with a novel BICD2 mutation | Kichula 2016 |  |
| 334 | Nosological delineation of congenital ocular motor apraxia type Cogan: An observational study | 10.1186/s13023-016-0486-z | Wente 2016 |
| 335 | Functional connectivity in the mouse brain during transitions from awake to deep anesthesia | 10.1177/0271678X16639009 | Bauer 2016 |
| 336 | Joubert's syndrome: Clinical and radiological findings in 14 patients | 10.1111/ene.13093 | AliPacha 2016 |
| 337 | An ominous cause of gum pain and fever in a young male. A case of burkitt's leukemia..ie. burkitt's lymphoma involving the bone marrow (bm) |  | Kaur 2016 |
| 338 | A lot is missing: the Orcade study | 10.1016/j.sleep.2015.10.008 | Quo 2016 |
| 339 | Joubert syndrome with autism in two siblings: A rare presentation | 10.4103/0019-5545.174395 | Raghavan 2016 |
| 340 | Rare disease: Lobar holoprosencephaly with a median cleft lip-Case report | 10.1597/14-087 | Radojicic 2016 |
| 341 | Odontogenic sinusitis resulting in abscess formation within the optic chiasm and tract: Case report and review | 10.1097/WNO.  0000000000000430 | Ghobrial 2016 |
| 342 | Anaesthesia and orphan disease: Sedation with ketofol in two patients with Joubert syndrome | 10.1097/EJA.0000000000000489 | Atalay 2016 |
| 343 | Migration of titanium cable into spinal cord and spontaneous C2 and C3 fusion: Case report of possible causes of fatigue failure after posterior atlantoaxial fixation | 10.1097/MD.0000000000005744 | Li 2016 |
| 344 | Joubert syndrome: A report of two cases | 10.1111/cga.12135 | Sameshima 2015 |
| 345 | An infant with abnormal breathing pattern | Gokdemir 2015 |  |
| 346 | Herpes simplex virus encephalitis in pregnancy | 10.1515/jpm-2015-2003 | Izquierdo 2015 |
| 347 | Neonatal incontinence pigmenti appearance with and without neurological involvement | 10.1515/jpm-2015-2003 | PriegoRuiz 2015 |
| 348 | Joubert syndrome in three siblings | 10.1007/s00467-015-3158-7 | Kf 2015 |
| 349 | Influence of trigeminal nerve lesion on facial growth: study of two cases of Goldenhar syndrome | 10.1051/orthodfr/2015013 | Darris 2015 |
| 350 | A classical syndrome and a revisited etiology: A patient with foix-chavany-marie Syndrome and radiotherapy induced vasculopathy | 10.1111/ene.12808 | Barbosa 2015 |
| 351 | Isolated lingual dyskinesias in multiple sclerosis |  | Park 2015 |
| 352 | Pituitary apoplexy: A rare clinical presentation of rhabdo-myolysis and altered mental status |  | Jimenez 2015 |
| 353 | Headache and Diplopia after Rapid Maxillary Expansion: A Clue to Underdiagnosed Pseudotumor Cerebri Syndrome? | 10.1055/s-0035-1555150 | Romeo 2015 |
| 354 | Update on neuroimaging phenotypes of mid-hindbrain malformations | 10.1007/s00234-014-1431-2 | Jissendi-Tchofo 2015 |
| 355 | Atlantoaxial instability of inflammatory origin in adults: Case reports, literature review, and rationale for early surgical intervention | 10.1227/NEU.  0000000000000578 | Kerolus 2015 |
| 356 | Pontine tegmental cap dysplasia: Report of two new cases from Kuwait | 10.1016/j.ejpn.2014.08.005 | Jovanovic 2015 |
| 357 | Association of Joubert Syndrome and Hirschsprung disease | 10.1007/s13312-015-0568-3 | Purkait 2015 |
| 358 | Correlation of hypothalamic activation with malocclusion: An fMRI study |  | Otsuka 2015 |
| 359 | Nanodiamond-mediated drug delivery and imaging: Challenges and opportunities | 10.1517/  17425247.2015.992412 | Vaijayanthimala 2015 |
| 360 | Hypomyelination, hypodontia, hypogonadotropic hypogonadism (4H) syndrome with vertebral anomalies: A novel association | 10.1177/0883073814541470 | Muthusamy 2015 |
| 361 | A case series of joubert syndrome |  | NandaKumar 2014 |
| 362 | Rare case of ataxia and developmental delay in girl child-joubert syndrome |  | Rathi 2014 |
| 363 | Case report: Ultrasound & MRI diagnosis of Joubert syndrome | 10.3109/  14767058.2014.924236 | Cassis-Martinez 2014 |
| 364 | A case of speech-induced oropharyngeal dystonia: A rare functional disorder |  | Hagiwara 2014 |
| 365 | Imaging of posterior fossa malformations | 10.1007/s00247-014-2968-2 | Rossi 2014 |
| 366 | The bishop's crook sign: A New MRI and neurosonography pareidolia in joubert syndrome | 10.1111/jon.12109 | Manley 2014 |
| 367 | Orofacial pain management: Current perspectives | 10.2147/JPR.S37593 | Romero-Reyes 2014 |
| 368 | The characteristic clinical symptoms of C-4 radiculopathy caused by ossification of the posterior longitudinal ligament: Case report | 10.3171/2014.2.SPINE13500 | Katsumi 2014 |
| 369 | The molar tooth sign is pathognomonic for joubert syndrome! | 10.1016/  j.pediatrneurol.2013.11.003 | Poretti 2014 |
| 370 | X-linked Joubert syndrome: Neuroimaging and clinical features associated with a novel mutation in oral-facial-digital syndrome type 1 | 10.3233/JPN-140657 | Hashemi 2014 |
| 371 | An unusual case of Ataxia: Joubert syndrome |  | Kumari 2013 |
| 372 | Prolonged daytime dissociative nrem3 sleep state in hypersomnolence with kleine levin symptomatology, a case report | 10.1016/j.sleep.2013.11.637 | Schreuder 2013 |
| 373 | The floppy newborn | 10.1016/S0378-3782(13)70110-5 | Orcesi 2013 |
| 374 | Removal of stimulus-induced artifacts in functional spinal cord imaging | 10.1177/1550059413507209 | Watanabe 2013 |
| 375 | Cavum septum pellucidum and cavum vergae with late-onset catatonia | 10.1097/YCT.0b013e318290fc13 | Yasaki 2013 |
| 376 | Focal dermal hypoplasia (goltz-gorlin syndrome): A new case with a novel variant in the PORCN gene (c.1250T>C:p.F417S) and unusual spinal anomaly | 10.1002/ajmg.a.35964 | Garavelli 2013 |
| 377 | Acro-osteolysis, keloid like-lesions, distinctive facial features, and overgrowth: Two newly recognized patients with premature aging syndrome, penttinen type | 10.1002/ajmg.a.35984 | Zufferey 2013 |
| 378 | Cervico-oculo-acoustic (Wildervanck) syndrome: Clinicoradiological findings | 10.1136/bcr-2013-009065 | Rihani 2013 |
| 379 | HR MRI characteristics of cerebellar abnormalities in children with Joubert's syndrome | 10.1007/s00247-013-2675-4 | Alikhanov 2013 |
| 380 | Improvement of post-traumatic segmental dystonia after correction of improperly placed dental implant | 10.1002/mds.25605 | RodriguezCruz 2013 |
| 381 | Joubert syndrome: A case report | 10.1007/s00415-013-6924-0 | AkgÃ¼n 2013 |
| 382 | Long-term outcome of bilateral pallidal deep brain stimulation in patients with primary | 10.1159/000351990 | Dystonia 2013 |
| 383 | Mirror movements in two hemiplegic cerebral palsy patients due to porencephaly | 10.4274/tftr.24.59.1 | Kara 2013 |
| 384 | Head nodding-differential diagnostic main sign | 10.1055/s-0033-1337698 | Baumgartner 2013 |
| 385 | Review of clinical presentation and diagnosis of mucopolysaccharidosis IVA | 10.1016/j.ymgme.2013.04.002 | Hendriksz 2013 |
| 386 | Familial Joubert syndrome in two siblings |  | MuhammadUsman 2012 |
| 387 | Temporomandibular disorders and occlusion | PMID: 23339268 | Badel 2012 |
| 388 | The cerebral representation of temporomandibular joint occlusion and its alternation by occlusal splints | 10.1002/hbm.21466 | Lotze 2012 |
| 389 | Temporomandibular joint growth adaptation and articular disc positional changes in functional orthopedic treatment: Magnetic resonance imaging investigation | PMID: 23961629 | Wangsrimongkol 2012 |
| 390 | Joubert syndrome and related disorders | 10.5114/ninp.2012.29565 | Paprocka 2012 |
| 391 | Maternal intrachromosomal insertional translocation leads to recurrent 1q21.3q23.3 deletion in two siblings | 10.1002/ajmg.a.35563 | Quinonez 2012 |
| 392 | Two cases of joubert syndrome with end stage renal failure | 10.1007/s00467-012-2232-7 | Yilmaz 2012 |
| 393 | Clinically mild form of joubert syndrome-related disorder in a 7-year-old female: A case report |  | Imataka 2012 |
| 394 | Avoiding CT scans in children with single-suture craniosynostosis | 10.1007/s00381-012-1721-0 | Schweitzer 2012 |
| 395 | Innovations in PET/CT | PMID: 22695337 | Klausen 2012 |
| 396 | Joubert syndrome with ocular defect in a Tunisian baby | 10.1111/j.  1469-8749.2012.04283.x | Abdelmoula 2012 |
| 397 | Diffusion tensor imaging and magnetic resonance spectroscopy of transient cerebral white matter lesions in X-linked Charcot-Marie-Tooth disease | 10.1016/j.jns.2012.01.017 | Sato 2012 |
| 398 | Joubert syndrome: Brain and spinal cord malformations in genotyped cases and implications for neurodevelopmental functions of primary cilia | 10.1007/s00401-012-0951-2 | Juric-Sekhar 2012 |
| 399 | A case of posterior spinal artery syndrome in the cervical cord: A review of the clinicoradiological literature | 10.2169/internalmedicine.  51.6922 | Murata 2012 |
| 400 | The â€œred dotâ€ on fa color maps: Clinical/anatomical correlation in malformations of the mid-hindbrain using DTI MR in children | 10.1007/s00247-012-2356-8 | Merlini 2012 |
| 401 | Preface | 10.1016/j.aanat.2011.09.003 | FanghÃ¤nel 2012 |
| 402 | Brain regional glucose uptake changes in isolated cerebellar cortical dysplasia: Qualitative assessment using coregistrated FDG-PET/MRI | 10.1007/s12311-011-0309-7 | Jissendi-Tchofo 2012 |
| 403 | Molar tooth sign | 10.1111/j.1440-  1754.2012.02514_1.x | Ghosh 2012 |
| 404 | Parameters of care for craniosynostosis | 10.1597/11-138 | McCarthy 2012 |
| 405 | Skeletal muscle MRI magnetisation transfer ratio reflects clinical severity in peripheral neuropathies | 10.1136/jnnp.2011.246116 | Sinclair 2012 |
| 406 | Breathing instability in Joubert syndrome | 10.1002/mds.23999 | Fabbri 2012 |
| 407 | Analysis of ascending spinal tract degeneration in cervical spondylotic myelopathy using 3D anisotropy contrast single-shot echo planar imaging on a 3.0-T system: Clinical article | 10.3171/2011.7.SPINE10843 | Urakawa 2011 |
| 408 | MRI shows increased sciatic nerve cross sectional area in inherited and inflammatory neuropathies | 10.1136/jnnp.2010.211334 | Sinclair 2011 |
| 409 | ALS and body movements - Compensation in higher order processing areas | 10.3109/17482968.2011.615522 | Heimrath 2011 |
| 410 | Association between prior alcohol use disorders and decreased prefrontal gray matter volumes in bipolar I disorder patients | 10.1016/j.neulet.2011.08.026 | Nery 2011 |
| 411 | Cerebral activation during unilateral clenching in patients with temporomandibular joint synovitis and biting pain: An functional magnetic resonance imaging study | 10.3760/cma.j.issn.  0366-6999.2011.14.011 | Zhao 2011 |
| 412 | A novel GJB1 mutation in an Italian patient with severe CMT and pyramidal signs | 10.1111/j.1529-  8027.2011.00316.x | Luigetti 2011 |
| 413 | Chronic bilirubin encephalopathy continues to occur in Canada |  | Sgro 2011 |
| 414 | Ataxic breathing in Joubert syndrome | 10.1002/mds.23764 | Fabbri 2011 |
| 415 | VIM DBS for irregular ataxic hand opening-closing action tremors in a patient with neuropathy and agenesis of the corpus callosum | 10.1002/mds.23764 | Ellias 2011 |
| 416 | 4h syndrome, dento-leukoencephalopathy or a new syndrome? | 10.1136/adc.2011.212563.63 | Sinha 2011 |
| 417 | Concurrence of dystonia 1 and Charcot-Marie-Tooth Neuropathy, type 1 A, in a large family | 10.1002/mds.23437 | Zirn 2011 |
| 418 | Molar tooth sign with ataxia and see-saw nystagmus (Joubert syndrome) | 10.4103/0972-2327.78057 | Byju 2011 |
| 419 | A report of Joubert syndrome in an infant, with literature review | 10.4103/1817-1745.84407 | Singh 2011 |
| 420 | Identification of signal bias in the variable flip angle method by linear display of the algebraic ernst equation | 10.1002/mrm.22849 | Helms 2011 |
| 421 | Brain activations in response to vibrotactile tooth stimulation: A psychophysical and fMRI study | 10.1152/jn.00565.2010 | Trulsson 2010 |
| 422 | Rhinocerebral mucormycosis as a cause of cerebral venous thrombosis | 10.1111/j.1747-  4949.2010.00480.x | Kajtazi 2010 |
| 423 | The liver disease in joubert syndrome related disorders (JSRD) | 10.1016/S1590-8658(10)60661-5 | Sciveres 2010 |
| 424 | Two cases of Joubert syndrome |  | Chandrasekaran 2010 |
| 425 | Clinical features and genetics of Joubert syndrome | 10.1007/s11825-010-0233-0 | Hellenbroich 2010 |
| 426 | Effects of toothbrushing on brain regional activities - A fMRI study | 10.1016/j.neures.2010.07.1960 | Mizuno 2010 |
| 427 | Trigemino-oculomotor synkinesis with oculo-oculomotor synkinesis caused diplopia | 10.1111/j.1468-  1331.2010.03233.x | KaradaÅŸ 2010 |
| 428 | Pontine tegmental cap dysplasia: Case report of a â€œnewâ€ disorder with associated cortical malformation | 10.1055/s-0030-1265588 | Weiss 2010 |
| 429 | Presentation of 16 paediatric patients manifesting with stroke during 2006-2010 | 10.1055/s-0030-1265612 | Brunner-Krainz 2010 |
| 430 | Chronic bilirubin encephalopathy: diagnosis and outcome | 10.1016/j.siny.2009.12.004 | Shapiro 2010 |
| 431 | Asymmetric oro-facial apraxia in primary lateral sclerosis - A perplexing clinical manifestation | 10.1007/s00415-010-5575-7 | PitaLobo 2010 |
| 432 | New Case of 4H Syndrome and a Review of the Literature | 10.1016/j.pediatrneurol.2010.01.015 | Orcesi 2010 |
| 433 | Dysplasia of the orbit and adjacent bone associated with plexiform neurofibroma and ocular disease in 42 NF-1 patients | PMID: 20592374 | Friedrich 2010 |
| 434 | Joubert syndrome: Unusual cause of neonatal distress | 10.3109/14767051003802503 | Carmona 2010 |
| 435 | Hemispheric prevalence during chewing in normal right-handed and left-handed subjects: a functional magnetic resonance imaging preliminary study. | 10.1179/crn.2010.016 | Bracco 2010 |
| 436 | Role of central leptin signaling in renal macrophage infiltration | 10.1507/endocrj.K09E-296 | Tanaka 2010 |
| 437 | Pathological and radiographic correlates of post-surgical inflammatory neuropathy | 10.1002/ana.22175 | Staff 2010 |
| 438 | Seckel syndrome with asymptomatic tonsillar herniation and congenital mirror movements | 10.1177/0883073809332694 | Thapa 2010 |
| 439 | Guidelines for pre-operative cardiac risk assessment and perioperative cardiac management in non-cardiac surgery: The task force for preoperative cardiac risk assessment and perioperative cardiac management in non-cardiac surgery of the European society o | 10.1097/EJA.  0b013e328334c017 | Poldermans 2010 |
| 440 | Enhancing effect of toothbrushing on neurocognition in the elderly | 10.1016/j.neures.2009.09.638 | Mizuno 2009 |
| 441 | Anterior genu corpus callosum and impulsivity in suicidal patients with bipolar disorder | 10.1016/j.neulet.2009.11.047 | Matsuo 2010 |
| 442 | Modeling the influence of TR and excitation flip angle on the Magnetization Transfer Ratio (MTR) in human brain Obtained from 3D spoiled gradient echo MRI | 10.1002/mrm.22379 | Helms 2010 |
| 443 | Joubert syndrome and related disorders: Report of five Tunisian cases | 10.3233/JPN-2010-0382 | Kraoua 2010 |
| 444 | Post-surgical inflammatory neuropathy | 10.1093/brain/awq252 | Staff 2010 |
| 445 | Safe MRI practice for physician |  | Giri 2010 |
| 446 | 'Molar tooth' sign in Joubert syndrome | 10.1007/s00247-009-1338-y | Mallick 2010 |
| 447 | Spatiotemporal complexity in the haemodynamic response to somatosensory stimulation in the un-anaesthetized rat | 10.1038/jcbfm.2009.133 | Martin 2009 |
| 448 | Linkage analysis and mutation screening in PANK2 and PLA2G6 genes in 7 consanguineous Saudi Arabian families with Karak syndrome |  | Azzedine 2009 |
| 449 | Anterior cingulate volumes associated with trait impulsivity in individuals with bipolar disorder | 10.1111/j.1399-  5618.2009.00732.x | Matsuo 2009 |
| 450 | Lobar holoprosencephaly with a median cleft: Case report | 10.1597/08-059.1 | Gawrych 2009 |
| 451 | Joubert Syndrome: Insights Into Brain Development, Cilium Biology, and Complex Disease | 10.1016/j.spen.2009.06.002 | Doherty 2009 |
| 452 | Development of fetal eye movements assessed by MRI | 10.1007/s00234-009-0561-4 | Woitek 2009 |
| 453 | Neuronal correlates of brain-derived neurotrophic factor Val66Met polymorphism and morphometric abnormalities in bipolar disorder | 10.1038/npp.2009.23 | Matsuo 2009 |
| 454 | Age-related peridural hyperemia in craniosynostotic rabbits | 10.1007/s00381-009-0812-z | Foley 2009 |
| 455 | Stereotactic radiosurgery with an upper partial denture | 10.2302/kjm.58.120 | Tayama 2009 |
| 456 | Myelin content abnormalities in the cerebellum in adult bipolar patients |  | Monkul 2009 |
| 457 | A voxel-based morphometry study of frontal gray matter correlates of impulsivity | 10.1002/hbm.20588 | Matsuo 2009 |
| 458 | Orbitofrontal cortex gray matter volumes in bipolar disorder patients: A region-of-interest MRI study | 10.1111/j.1399-  5618.2009.00662.x | Nery 2009 |
| 459 | Neuroleptic malignant syndrome due to risperidone treatment in a child with joubert syndrome | 10.1016/S0027-  9684(15)30858-0 | Vurucu 2009 |
| 460 | Intraneural perineurioma - A rare cause of mononeuropathy in childhood | 10.1111/j.1469-  8749.2008.03208.x | Mccelland 2009 |
| 461 | Orbitofrontal cortex volumes in medication naÃ¯ve children with major depressive disorder: A magnetic resonance imaging study | 10.1089/cap.2008.053 | Chen 2008 |
| 462 | Neuroplasticity of edentulous patients with implant-supported full dentures | 10.1111/j.1600-  0722.2008.00557.x | Yan 2008 |
| 463 | Illness duration and total brain gray matter in bipolar disorder: Evidence for neurodegeneration? | 10.1016/j.  euroneuro.2008.04.015 | Frey 2008 |
| 464 | Abnormal corpus callosum myelination in pediatric bipolar patients | 10.1016/j.  jad.2007.10.006 | Caetano 2008 |
| 465 | Oculocerebrocutaneous syndrome | 10.3928/01913913-  20080501-19 | Saatci 2008 |
| 466 | MRI study of the cerebellum in young bipolar patients | 10.1016/j.pnpbp.2007.09.016 | Monkul 2008 |
| 467 | Striatal volume abnormalities in treatment-naÃ¯ve patients diagnosed with pediatric major depressive disorder | 10.1089/cap.2007.0026 | Matsuo 2008 |
| 468 | Brain abscess secondary to dental braces | 10.1097/INF.  0b013e31815819a6 | Wolf 2008 |
| 469 | Prefrontal gray matter increases in healthy individuals after lithium treatment: A voxel-based morphometry study | 10.1016/j.neulet.  2007.09.074 | Monkul 2007 |
| 470 | Where in the brain is wrong with the congenital ocular motor apraxia |  | Chuman 2007 |
| 471 | Medial temporal lobe abnormalities in pediatric unipolar depression | 10.1016/j.neulet.2007.06.014 | Caetano 2007 |
| 472 | MRI study of corpus callosum in patients with borderline personality disorder. A pilot study | 10.1016/j.pnpbp.2007.07.013 | Zanetti 2007 |
| 473 | Insertion of discrete phonological units: An articulatory and acoustic investigation of aphasic speech | 10.1080/01690960701273532 | Buchwald 2007 |
| 474 | Congenital ocular motor apraxia: Clinical and neuroradiological findings, and long-term intellectual prognosis | 10.1016/j.braindev.2007.01.002 | Kondo 2007 |
| 475 | Meckel syndrome in the Hutterite population is actually a Joubert-related cerebello-oculo-renal syndrome | 10.1002/ajmg.a.31832 | Boycott 2007 |
| 476 | The importance of dental-based treatment shown on the case report of a pontine abscess caused by Streptococcus viridans | 10.1007/s10006-007-0053-9 | MÃ¼ller-Richter 2007 |
| 477 | Joubert syndrome: Description, diagnosis and guidance |  | FransenVanDePutte 2007 |
| 478 | Malignant transformation of a lumbar intradural dermoid cyst | 10.1007/s00776-007-1125-2 | Kudo 2007 |
| 479 | Fronto-limbic brain structures in suicidal and non-suicidal female patients with major depressive disorder | 10.1038/sj.mp.4001919 | Monkul 2007 |
| 480 | The molar tooth sign of Joubert syndrome | 10.1001/archneur.64.4.602 | Kumar 2007 |
| 481 | Anatomical measurements of the orbitofrontal cortex in child and adolescent patients with bipolar disorder | 10.1016/j.neulet.2006.10.016 | Najt 2007 |
| 482 | Prefrontal hyperactivation during working memory task in untreated individuals with major depressive disorder | 10.1038/sj.mp.4001894 | Matsuo 2007 |
| 483 | Joubert syndrome: Review and report of five cases from India | 10.1055/s-0035-1557400 | Singhi 2007 |
| 484 | Skull base chordoma mimicking a preauricular neoplasm in a child: Clinicopathological features and biological behaviour | 10.1016/j.jcms.2006.10.001 | Suba 2007 |
| 485 | New syndromic form of benign hereditary chorea is associated with a deletion of TITF-1 and PAX-9 contigous genes | 10.1002/mds.21135 | Devos 2006 |
| 486 | MRI study of thalamus volumes in juvenile patients with bipolar disorder | 10.1002/da.20161 | Monkul 2006 |
| 487 | Testing causal mechanisms of nonsyndromic craniosynostosis using path analysis of cranial contents in rabbits with uncorrected craniosynostosis | 10.1597/05-107 | Fellows-Mayle 2006 |
| 488 | Eye movements of Charcot-Marie-Tooth diseases | 10.3757/jser.65.245 | Sugasawa 2006 |
| 489 | Smaller cingulate volumes in unipolar depressed patients | 10.1016/j.biopsych.  2005.10.011 | Caetano 2006 |
| 490 | Joubert Syndrome and "molar sign" in the renal-ocular-cerebelar complex in two patients | 10.4067/S0370-41062005000600008 | Bibas 2005 |
| 491 | Diferential diagnosis of temporomandibular joint disorders | PMID: 16264383 | Bermejo-Fenoll 2005 |
| 492 | Clinicopathological aspects of the neuropathy of neurogastrointestinal encephalomyopathy (MNGIE) in four patients including two with a Charcot-Marie-Tooth presentation | 10.1007/s00415-005-0712-4 | Said 2005 |
| 493 | Age-related changes in lateral ventricle morphology in craniosynostotic rabbits using magnetic resonance imaging | 10.1007/s00381-004-1107-z | Fellows-Mayle 2005 |
| 494 | Myoclonus and generalized digestive dysmotility in triple A syndrome with AAAS gene mutation | 10.1002/mds.10660 | Roubergue 2004 |
| 495 | Vermian hypoplasia and arrested cerebral myelination in two sisters: Variant of Joubert's syndrome or a new syndrome? | 10.1177/08830738030180111001 | DeMyer 2003 |
| 496 | Functional magnetic resonance imaging of human jaw movements. | 10.1046/j.1365-2842.2003.01054.x | Tamura 2003 |
| 497 | Clinical and genetic aspects of the Joubert syndrome: A disorder characterised by cerebellar vermian hypoplasia and accompanying brainstem malformations | 10.2174/1389202033350083 | Bennett 2003 |
| 498 | Is tooth agenesis related to brainstem anomalies in myelomeningocele patients with Chiari II malformations? | 10.1080/000163502762667351 | LinderstrÃ¶m 2002 |
| 499 | Joubert syndrome: Radiological features in MRI | 10.1007/s00062-002-3336-4 | Rachinger 2002 |
| 500 | Two cases of oral dyskinesia successfully treated with brief psychotherapy,drug therapy and local stimulation therapy |  | Mashu 2001 |
| 501 | Joubert syndrome: Clinical and radiological observations |  | Kentab 2001 |
| 502 | A rabbit model of human familial, nonsyndromic unicoronal suture synostosis. II. Intracranial contents, intracranial volume, and intracranial pressure | 10.1007/s003810050220 | Mooney 1998 |
| 503 | 'Joubert syndrome' revisited: Key ocular motor signs with magnetic resonance imaging correlation | 10.1177/  088307389701200703 | Maria 1997 |
| 504 | A 56 year old man with fever, backache, and tetraparesis |  | Hattori 1996 |
| 505 | Comparison of cerebral activity during teeth clenching and fist clenching: a functional magnetic resonance imaging study | 10.1111/j.1600-  0722.2010.00784.x | Iida 2010 |
| 506 | Analysis of brain and muscle activity during low-level tooth clenching - a feasibility study with a novel biting device | 10.1111/joor.12128 | Iida 2014 |
| 507 | An fMRI Study of the Brain Network Involved in Teeth Tapping in Elderly Adults | 10.3389/fnagi.2020.00032 | Kobayashi 2020 |
| 508 | Preliminary research on activating cerebral cortex with premature contact on tooth | IJCEM0064716 | Yu 2018 |
| 509 | Principles of the magnetic resonance imaging movie method for articulatory movement | 10.1007/s11282-018-0347-7 | Yoshida 2019 |
| 510 | Tongue Posture, Tongue Movements, Swallowing, and Cerebral Areas Activation: A Functional Magnetic Resonance Imaging Study | 10.3390/app10176027 | Scoppa 2020 |
| 511 | Imaging of temporomandibular joint abnormalities in juvenile idiopathic arthritis with a focus on developing a magnetic resonance imaging protocol | 10.1007/s00247-017-4005-8 | Miller 2018 |
| 512 | Sonographic 'molar tooth' sign in the diagnosis of Joubert syndrome | 10.1002/uog.8979 | Pugash 2011 |
| 513 | Brain alterations in sensorimotor and emotional regions associated with temporomandibular disorders | 10.1111/odi.14466 | Yin 2023 |
| 514 | High-resolution magnetic resonance imaging and diffusion tensor imaging of the porcine temporomandibular joint disc | 10.1259/dmfr/19195745 | Benavides 2009 |
| 515 | Influence of periodontal afferent inputs for human cerebral blood oxygenation during jaw movements | 10.1007/s00221-011-2941-3 | Iida 2012 |
| 516 | Magnetic resonance imaging artefacts and fixed orthodontic attachments | 10.1093/ejo/cju020 | Beau 2015 |
| 517 | Preliminary Study Evaluating the Accuracy of MRI Images on CBCT Images in the Field of Orthodontics | 10.17796/jcpd.  36.2.r7853hp574045414 | Tai 2011 |
| 518 | Classification of mouth movements using 7T fMRI | 10.1088/1741-  2560/12/6/066026 | Bleichner 2015 |
| 519 | Influence of orthodontic brackets and permanent retainers on the diagnostic image quality of MRI scans: A preliminary study | 10.17219/dmp/132390 | Neela 2021 |
| 520 | An fMRI study on the effects of jaw-tapping movement on memory function in elderly people with memory disturbances | 10.1016/j.eujim.2013.10.004 | Cho 2014 |
| 521 | Comparison of cerebral activation involved in oral and manual stereognosis | 10.1016/j.jocn.2011.03.005 | Fujii 2011 |
| 522 | SHIP-MR and Radiology: 12 Years of Whole-Body Magnetic Resonance Imaging in a Single Center | 10.3390/healthcare10010033 | Hosten 2022 |
| 523 | ORTHODONTIC APPLIANCES AND MAGNETIC-RESONANCE IMAGING OF THE BRAIN AND TEMPOROMANDIBULAR-JOINT | DOI: 10.1043/0003-3219(1988)058<0009:OAAMRI>2.0.CO;2 | SADOWSKY 1988 |
| 524 | Preferred chewing side-dependent two-point discrimination and cortical activation pattern of tactile tongue sensation | 10.1016/j.bbr.2009.04.028 | Minato 2009 |
| 525 | Molar tooth sign of the midbrain-hindbrain junction: Occurrence in multiple distinct syndromes | 10.1002/ajmg.a.20437 | Gleeson 2004 |
| 526 | The Temporal Muscle of the Head Can Cause Artifacts in Optical Imaging Studies with Functional Near-Infrared Spectroscopy | 10.3389/fnhum.2017.00456 | Schecklmann 2017 |
| 527 | Effects of orthodontic appliances on the diagnostic capability of magnetic resonance imaging in the head and neck region: A systematic review | 10.1016/j.ortho.2019.06.001 | Hasanin 2019 |
| 528 | Autosomal recessive spastic ataxia of Charlevoix Saguenay (ARSACS): expanding the genetic, clinical and imaging spectrum | 10.1186/1750-1172-8-41 | Synofzik 2013 |
| 529 | Genetic defects disrupting glial ion and water homeostasis in the brain | 10.1111/bpa.12602 | Min 2018 |
| 530 | Differential cerebral activation during observation of expressive gestures and motor acts | 10.1016/j.neuropsychologia.  2006.03.016 | Lotze 2006 |
| 531 | Semi-automatic segmentation for 3D motion analysis of the tongue with dynamic MRI | 10.1016/j.  compmedimag.2014.07.004 | Lee 2014 |
| 532 | Application of in vivo magnetic resonance imaging to define the pathological characteristics of excitotoxic spinal cord injury |  | Berens 2003 |
| 533 | fMRI Study of the Effects of Visual Feedback Manipulation on Sensorimotor Circuits | 10.1109/NEBC.2010.5458226 | IEEE 2010 |
| 534 | Motor coordination of articulators depends on the place of articulation | 10.1016/j.bbr.2008.12.008 | Inoue-Arai 2009 |
| 535 | Joubert Syndrome Associated with Severe Central Sleep Apnea | PMID: 20726289 | Wolfe 2010 |
| 536 | Brain perfusion impairment in neurologically asymptomatic adult patients with sickle-cell disease shown by voxel-based analysis of SPECT images | 10.3389/fneur.2013.00207 | Deus-Silva 2013 |
| 537 | Magnets in medicine | 10.1179/026708301125000140 | Riley 2002 |
| 538 | Neural representations of skilled movement | 10.1093/brain/123.11.2306 | Haaland 2000 |
| 539 | Septic Cavernous Sinus Thrombosis Caused by Dental Infection | 10.3341/jkos.2022.63.5.467 | Kim 2022 |
| 540 | Acoustic radiation force contrast in MRI: Detection of calcifications in tissue-mimicking phantoms | 10.1118/1.3512806 | Mende 2010 |
| 541 | Reaffirming the link between chronic phantom limb pain and maintained missing hand representation | 10.1016/j.cortex.2018.05.013 | Kikkert 2018 |
| 542 | Brief Report: Approaches to P-31-MRS in Awake, Non-Sedated Children With and Without Autism Spectrum Disorder (vol 42, pg 1120, 2012) | 10.1007/s10803-013-1821-z | Erickson 2014 |
| 543 | LEBERS PLUS - NEUROLOGICAL ABNORMALITIES IN PATIENTS WITH LEBERS HEREDITARY OPTIC NEUROPATHY | 10.1136/jnnp.59.2.160 | NIKOSKELAINEN 1995 |
| 544 | Controlling jaw-related motion artifacts in functional near-infrared spectroscopy | 10.1016/j.jneumeth.  2023.109810 | Zhang 2023 |
| 545 | Wavelet Transform in Biomedical Image Segmentation and Classification |  | Prochazka 2011 |
| 546 | Proton magnetic resonance spectroscopy of the thalamus in patients with chronic neuropathic pain after spinal cord injury | PMID: 12063213 | Pattany 2002 |
| 547 | A new and fast approach towards sEMG decomposition | 10.1007/s11517-012-1029-y | Gligorijevic 2013 |
