# Supplementary material for: Systematic review on effects of experimental orthodontic tooth displacement on brain activation assessed by fMRI
Source: Clin Exp Dent Res. 2024 Apr 1;10(2):e879. doi: 10.1002/cre2.879 (PMC10982672; doi:10.1002/cre2.879)
Supplement: Supplementary file 3 — Supplementary information. [file CRE2-10-e879-s004.docx]

**Appendix 2: Electronic Search Strategy Updated on September 28^th^, 2023**

| **Database** | **Search Strategy (key terms)** | **Results** |
| --- | --- | --- |
| PubMed | #1: "brain"[MeSH Terms] OR "brain"[All Fields] OR "cerebrum"[MeSH Terms] OR "cerebrum"[All Fields] OR "central nervous system"[MeSH Terms] OR "central nervous system"[All Fields] OR "CNS"[All Fields]  #2: "orthodont*"[All Fields] OR orthodont*[MeSH Terms] OR "orthodontic force"[All Fields] OR "tooth movement"[All Fields] OR (("tooth"[MeSH Terms] OR "tooth"[All Fields]) AND "movement*"[All Fields]) OR tooth mobil*[All Fields] OR "dental orthopedic"[All Fields] OR ("dental"[All Fields] AND "orthopedic*"[All Fields]) OR "tooth retraction"[All Fields] OR (("tooth"[MeSH Terms] OR "tooth"[All Fields]) AND "retract*"[All Fields]) OR "tooth migration"[All Fields] OR "tooth displacement"[All Fields]  #3: "magnetic resonance imaging"[MeSH Terms] OR "Magnetic Resonance Imaging"[All Fields] OR "MRI"[All Fields] OR "fMRI"[All Fields]  #4: #1 AND #2 AND #3 | 2,571,961  109,089  738,915  149 |
| [Cochrane Central Register of Controlled Trials](https://www.cochranelibrary.com/) (CENTRAL) | #1 (brain)  #2 (cerebrum)  #3 ("central nervous system")  #4 (CNS)  #5 (orthodont*)  #6 ("orthodontic force")  #7 ("tooth movement")  #8 (tooth AND movement)  #9 ("tooth mobility")  #10 ("dental orthopedic")  #11 (dental AND orthopedic*)  #12 ("tooth retrtaction")  #13 (tooth AND retraction)  #14 ("tooth migration")  #15 ("tooth displacement")  #16 ("magnetic resonance imaging")  #17 (MRI)  #18 (fMRI)  #19 #1 OR #2 OR #3 OR #4  #20 #5 OR #6 OR #7 OR #8 OR #9 OR #10 OR #11 OR #12 OR #13 OR #14 OR #15  #21 #16 OR #17 OR #18  #22 #19 AND #20 AND #21 | 82,567  166  14,583  6,822  6,548  96  976  1,470  302  9  762  0  504  19  15  31,956  33,250  5,863  96,281  7,491  44,846  14 |
| EMBASE | #1: 'brain' OR 'cerebrum' OR 'central nervous system' OR 'CNS'  #2: orthodont* OR 'orthodontic force' OR 'tooth movement' OR (tooth AND movement*) OR 'tooth mobility' OR 'dental orthopedic' OR (dental AND orthopedic*) OR ‘tooth retraction’ OR (tooth AND retraction) OR ‘tooth migration’ OR ‘tooth displacement’  #3: 'magnetic resonance imaging' OR ‘MRI' OR 'fMRI'  #4: #1 AND #2 AND #3 | 3,931,899  121,370  1,259,865  472 |
| Web of Science | (“brain” OR “cerebrum” OR “central nervous system” OR “CNS”) AND (orthodont* OR “orthodontic force” OR “tooth movement” OR (tooth AND movement*) OR “tooth mobility” OR “dental orthopedic” OR (dental AND orthopedic*) OR “tooth retraction” OR (tooth AND “retraction”) OR “tooth migration” OR “tooth displacement”) AND (“magnetic resonance imaging” OR “MRI” OR “fMRI”) | 156 |
