# Supplementary material for: Systematic review on effects of experimental orthodontic tooth displacement on brain activation assessed by fMRI
Source: Clin Exp Dent Res. 2024 Apr 1;10(2):e879. doi: 10.1002/cre2.879 (PMC10982672; doi:10.1002/cre2.879)
Supplement: Supplementary file 4 — Supplementary information. [file CRE2-10-e879-s002.docx]

**Appendix 4: Brain Regions Altered in Activation or FC**

| **Studies** | **Imaging**  **Modality** | **Analysis method** | **Correction for multiple comparisons** | **Content** | | | | | |
| --- | --- | --- | --- | --- | --- | --- | --- | --- | --- |
| Ariji et al. 2018 | fMRI | activated brain region BOLD whole-brain | cluster corrected  (*p* < 0.05) | **Regions with Significant increase in fMRI signal during insertion of the apparatuses minus baseline** | | | | | |
|  |  |  |  | **Region BA side** | **MNI [mm]**  **x y z** | | | **Maximum *T* value** | **Cluster size (voxels)** |
|  |  |  |  | **Brass contact gauge:** | | | | | |
|  |  |  |  | Parietal association area 5 L | 12 | -50 | 20 | 4.02 |  |
|  |  |  |  | 40 L | -26 | -50 | 46 | 3.31 |  |
|  |  |  |  | Frontal association area 44 L | -36 | 18 | 8 | 3.95 |  |
|  |  |  |  | Temporal association area 20 L | -46 | -50 | -22 | 3.73 |  |
|  |  |  |  | 21 L | -54 | -50 | 20 | 3.42 |  |
|  |  |  |  | 22 L | -48 | -46 | 18 | 3.66 |  |
|  |  |  |  | 37 L | -38 | -62 | -18 | 3.60 |  |
|  |  |  |  | Thalamus R | 22 | -24 | 4 | 3.44 |  |
|  |  |  |  | Hippocampus R | 22 | -8 | -12 | 3.63 |  |
|  |  |  |  | Putamen L | -26 | -16 | 8 | 3.96 |  |
|  |  |  |  | Lingual gyrus L | -10 | -74 | -10 | 4.00 |  |
|  |  |  |  | Calcarine sulcus R | 10 | -84 | 10 | 3.39 |  |
|  |  |  |  | Insula 13 L | -40 | -2 | 18 | 3.33 |  |
|  |  |  |  | Cerebellum L | -18 | -56 | -14 | 3.68 |  |
|  |  |  |  | **Floss:** | | | | | |
|  |  |  |  | Parietal association area 40 L | -48 | -40 | 26 | 4.31 |  |
|  |  |  |  | Frontal association cortex 11 L | -28 | 28 | -8 | 4.90 |  |
|  |  |  |  | 44 L | -46 | -28 | 20 | 3.59 |  |
|  |  |  |  | Temporal association area 20 L | -46 | -52 | -10 | 3.77 |  |
|  |  |  |  | 21 L | -52 | 2 | -24 | 3.58 |  |
|  |  |  |  | 22 L | -42 | -20 | 0 | 3.45 |  |
|  |  |  |  | 37 L | -42 | -54 | -18 | 4.14 |  |
|  |  |  |  | 38 L | -48 | 8 | -20 | 3.20 |  |
|  |  |  |  | Hippocampus L | -32 | -8 | -22 | 3.31 |  |
|  |  |  |  | Amygdala L | -30 | -2 | -18 | 3.36 |  |
|  |  |  |  | Insula 13 L | -34 | 12 | -8 | 3.84 |  |
|  |  |  |  | Cerebellum L | -20 | -46 | -20 | 3.62 |  |

Only significant clusters of activation corrected were listed (P < 0.05), which were carried out for multiple comparisons.

The maximal t value indicated the most significant peak activations in each cluster.

BA: Brodmann area, fMRI: functional magnetic resonance imaging, L: left, R: right, MNI: Montreal Neurological Institute.

| **Studies** | **Imaging**  **modality** | **Analysis method** | **Correction for multiple comparisons** | **Content** | | | | | |
| --- | --- | --- | --- | --- | --- | --- | --- | --- | --- |
| Ariji et al. 2019 | fMRI | activated brain region BOLD  whole-brain | no information is available | **Activated regions in the clenching group (Task 1)** | | | | | |
|  |  |  |  | **Low level clenching with 10% of maximal biting force** | | | | | |
|  |  |  |  | **Region BA side** | **MNI [mm]**  **x y z** | | | **Maximum *T* value** | **Cluster size (voxels)** |
|  |  |  |  | Supplementary motor area 6 L | -2 | -8 | 70 | 4.22 |  |
|  |  |  |  | R | 62 | 0 | 32 | 4.75 |  |
|  |  |  |  | Frontal association area 44-45 L | -60 | 16 | 20 | 6.35 |  |
|  |  |  |  | R | 56 | 4 | 6 | 4.17 |  |
|  |  |  |  | Cerebellum L | -18 | -90 | -20 | 3.67 |  |
|  |  |  |  | Striate and parastriate 17-18 L | -16 | -102 | -10 | 4.33 |  |
|  |  |  |  | R | 24 | -94 | -2 | 4.41 |  |
|  |  |  |  | **Low level clenching with 40% of maximal biting force** | | | | | |
|  |  |  |  | Primary sensorimotor cortex 1,4 L | -54 | -24 | 52 | 5.75 |  |
|  |  |  |  | R | 56 | 12 | 24 | 5.67 |  |
|  |  |  |  | Supplementary motor area 6 L | -50 | 0 | 38 | 4.88 |  |
|  |  |  |  | Frontal association area 44-45 L | -46 | 10 | 36 | 4.70 |  |
|  |  |  |  | Temporal association area 20-22,37 L | -60 | -46 | 10 | 4.36 |  |
|  |  |  |  | R | 58 | -68 | 0 | 5.32 |  |
|  |  |  |  | Cerebellum L | -36 | -50 | -44 | 4.39 |  |
|  |  |  |  | Visual cortex 17-18 L | -14 | -98 | 0 | 5.00 |  |
|  |  |  |  | R | 22 | -80 | -8 | 4.12 |  |
|  |  |  |  | **Activated regions in the tooth separation group (Task 2)** | | | | | |
|  |  |  |  | **Without biting** | | | | | |
|  |  |  |  | **Region BA side** | **MNI [mm]**  **x y z** | | | **Maximum *T* value** | **Cluster size (voxels)** |
|  |  |  |  | Primary sensorimotor cortex 2-4 L | -42 | 0 | 32 | 3.49 |  |
|  |  |  |  | Frontal association area 8,44,46 L | -42 | -24 | 2 | 3.33 |  |
|  |  |  |  | Temporal association area 2-22,37 L | -46 | -32 | -18 | 4.36 |  |
|  |  |  |  | Cerebellum L | -16 | -74 | -50 | 3.75 |  |
|  |  |  |  | R | 14 | -56 | -48 | 4.00 |  |
|  |  |  |  | **With biting** | | | | | |
|  |  |  |  | Primary sensorimotor cortex 2 L | -46 | -24 | 28 | 3.10 |  |
|  |  |  |  | Parietal association area 5,40 L | 12 | -50 | 22 | 3.73 |  |
|  |  |  |  | 40 R | 50 | -50 | 22 | 3.13 |  |
|  |  |  |  | Frontal association area 44 L | -38 | 18 | 8 | 4.04 |  |
|  |  |  |  | Temporal association area 20-22,37 L | -40 | -38 | 20 | 4.45 |  |
|  |  |  |  | Lingual gyrus L | -10 | -74 | -10 | 3.36 |  |
|  |  |  |  | R | 16 | -62 | 2 | 3.19 |  |
|  |  |  |  | Thalamus L | -22 | -10 | 14 | 3.49 |  |
|  |  |  |  | R | 12 | -24 | 0 | 3.39 |  |
|  |  |  |  | Hippocampus R | 22 | -8 | -12 | 3.58 |  |
|  |  |  |  | Putamen L | -26 | -16 | 4 | 4.26 |  |
|  |  |  |  | Insula 13 L | -38 | -18 | 4 | 3.46 |  |
|  |  |  |  | Cerebellum L | -18 | -52 | -14 | 3.58 |  |

Regions with significantly higher maximum voxel T values than those just after the gage insertion.

| **Studies** | **Imaging**  **modality** | **Analysis method** | **Correction for multiple comparisons** | **Content** | | | | | |
| --- | --- | --- | --- | --- | --- | --- | --- | --- | --- |
| Jin et al. 2021 | rs-fMRI  before and after the separator placement | fALFF-ROI/  seed-based-FC approach  ROI | Voxel level:  - FDR for fALFF analysis  (p < 0.05)  - FWE for FC analysis  (p < 0.05)  AlphaSim:  for covariates  (p < 0.05) | **Significant differences in regional fALFF between the orofacial pain caused by orthodontic separator and control groups** | | | | | |
|  |  |  |  | **Regions of thalamus showing increased fALFF in orofacial pain group relative to control group** | | | | | |
|  |  |  |  | **Region BA side** | **MNI [mm]**  **x y z** | | | ***T* value** | **Cluster size (voxels)** |
|  |  |  |  | Dorsal thalamus R | 9 | -21 | 12 | -7.89 | 68 |
|  |  |  |  | L | -12 | -27 | 12 | -5.43 | 35 |
|  |  |  |  | **Regions of thalamus showing decreased fALFF in orofacial pain group relative to control group** | | | | | |
|  |  |  |  | Medial thalamus R | 9 | -21 | 3 | 6.56 | 51 |
|  |  |  |  | L | -6 | -15 | 6 | 6.58 | 34 |
|  |  |  |  | **Correlations between the medial thalamus-seeded FC and VAS changes in the orofacial pain group** | | | | | |
|  |  |  |  | **Positive correlation** | | | | | |
|  |  |  |  | **Region BA side** | **MNI [mm]**  **x y z** | | | ***T* value** | **Cluster size (voxels)** |
|  |  |  |  | ACC R | 15 | 15 | 51 | 4.36 | 168 |
|  |  |  |  | Posterior cingulate cortex | -3 | -54 | 24 | 3.08 | 337 |
|  |  |  |  | **Negative correlation** | | | | | |
|  |  |  |  | Cerebellum | -34 | -54 | -45 | -4.11 | 166 |

*The fALFF of the medial thalamus was significantly decreased, and that of the dorsal area of the thalamus was significantly increased compared with those of the control group*

*(p* < *0.05, FDR corrected).*

| **Studies** | **Imaging**  **modality** | **Analysis method** | **Correction for multiple comparisons** | **Content** | | | | | |
| --- | --- | --- | --- | --- | --- | --- | --- | --- | --- |
| Maurer et al. 2021 | t-fMRI | activated brain region BOLD whole-brain | FWE corrected  (cluster corrected)  P < 0.001 | **Peak coordinates of the observed significant clusters derived from the paired t test T2 > T1** | | | | | |
|  |  |  |  | **Region BA side** | **MNI**  **x y z** | | | ***T* value** | **Cluster size (voxels)** |
|  |  |  |  | S1 L | -51 | -13 | 50 | NA | 1109 |
|  |  |  |  | Insula L | -31 | 5 | 11 | NA |  |
|  |  |  |  | M1 L | -42 | -19 | 59 | NA |  |
|  |  |  |  | SMA L | -9 | -4 | 53 | NA | 496 |
|  |  |  |  | SMA | 0 | 11 | 50 | NA |  |
|  |  |  |  | SMA R | 15 | -4 | 50 | NA |  |
|  |  |  |  | Insula R | 36 | -4 | 11 | NA | 1095 |
|  |  |  |  | Rolandic operculum R | 57 | -4 | 14 | NA |  |
|  |  |  |  | M1/S1 R | 51 | -7 | 50 | NA |  |

Peak coordinates of the observed significant clusters derived from the paired t-test painful tooth clenching (T2) > experimental tooth clenching (T1). Coordinates (x,y,z) are in MNI space. L: left, M1: primary motor cortex, R: right, S1: primary somatosensory cortex, SMA supplementary motor area.

| **Studies** | **Imaging**  **modality** | **Analysis method** | **Correction for multiple comparisons** | **Content** | | | | | |
| --- | --- | --- | --- | --- | --- | --- | --- | --- | --- |
| Yang et al. 2015 | rs-fMRI | Voxel-wise  ALFF/  seed-based-FC approach  ROI | FDR corrected  p < 0.05 | **ALFF differences between subjects In normal state and pain state induced with orthodontic Separator** | | | | | |
|  |  |  |  | **Region BA side** | **MNI**  **x y z** | | | ***T* value**  ***P* value** | **Cluster size (voxels)** |
|  |  |  |  | **P>C** | | | | | |
|  |  |  |  | Insula (Sub-lobar) L | -45 | -24 | 21 | 5.3172  P < 0.001 | 97 |
|  |  |  |  | Supp_Motor_Area (Frontal Lobe) R | 6 | -18 | 48 | 5.1273  P = 0.001 | 85 |
|  |  |  |  | **C>P** | | | | | |
|  |  |  |  | Pyramis (Cerebellum Posterior Lobe) L | -21 | -84 | -42 | 7.1469  P < 0.001 | 332 |
|  |  |  |  | Uvula (CerebaKum Posierior Lobe) R | 15 | -81 | -33 | 4.6208  P < 0.001 | 175 |
|  |  |  |  | Superior Frontal Gyrus (Frontal Lobe) L | -15 | 51 | 33 | 3.6913  P < 0.001 | 79 |
|  |  |  |  | Angular Gyrus (Parietal Lobe) R | 33 | -57 | 33 | 5.347  P < 0.001 | 133 |
|  |  |  |  | Angular Gyrus (Prscuneus) L | -33 | -63 | 36 | 5.0678  P < 0.001 | 128 |
|  |  |  |  | **Abnormal brain regions In subjects with orthodontic pain induced with orth. Separator by FC analysis** | | | | | |
|  |  |  |  | **Seed region connected region correlation** | **MNI**  **x y z** | | | **Z score**  **No Pain > Pain** | **Cluster size (voxels)** |
|  |  |  |  | IC.L (-45, -24, 21) CPLP.R -0.009 (PPI) | 15 | -84 | -33 | 6.87 | 27 |
|  |  |  |  | P.L (-33, -63, 36) MTG.L -0.005 (VAS) | -51 | -75 | 24 | 4.28 | 26 |
|  |  |  |  | PC.L(-21, -84, -42) PL.L / | -45 | -24 | 21 | -14.24 | 13 |
|  |  |  |  | COL / | 6 | -75 | 9 | -9.38 | 10 |
|  |  |  |  | **Seed region connected region correlation** | **MNI**  **x y z** | | | **Z score**  **Pain >**  **No Pain** | **Cluster size (voxels)** |
|  |  |  |  | PL.R (33. -57, 33) CPLPL -0.017 (PPI)  -0.016 (PPI) | -21 | -84 | -42 | 5.46 | 13 |

N =Normal. T and P values from a t-test of the peak voxel (showing greatest statistical difference within a cluster), which corresponds to a corrected P < 0.01. IC.L: left Insular cortex, P.L: left precuneus, CPLP.R: right cerebellum posterior lobe-pyramis, MTG.L: left middle temporal gyrus, PC.L: left posterior cerebellum, PL.L: left parietal lobe

| **Studies** | **Imaging**  **modality** | **Analysis method** | **Correction for multiple comparisons** | **Content** |
| --- | --- | --- | --- | --- |
| Zhang et al. 2020 | rs-fMRI | K-means clustering  Algorithm  BOLD  Whole-brain  FC | Bonferroni  correction  P < 0.05 | - Alternations in FC between GM networks: - increase: GM3 and GM5 and between GM5 and GM7; - decreased FC in: GM2-GM3-GM8  - Alternations in FC between WM networks: - increase: between WM12 and: WM1, WM4, WM14; - decreased : WM1-WM2-WM11-WM3-WM4-WM5-WM11 and WM5-WM12-WM9  - Alternations in FC between GM and WM networks: - increase: GM5 and WM4 , and GM6-WM9-GM7-WM2; - increase: WM12 and 9 GMs  - decreased: GM3 and WM5, between GM8 and WM11, and in WM1-GM10-WM2  - Alternation in FC in GM-WM loops: DMN-WM12-WM4-DMN, DAN-WM12-WM5-DAN, SN-WM12-WN9-SN |

lateral visual network (GM1), anterior lobe of cerebellum network (GM2), dorsal attention network (DAN) (GM3), medial occipital network (GM4), DMN (Default Mode Network) (GM5), superior frontal network (GM6), SN (GM7), executive control network (ECN) (GM8), somatomotor network (GM9), posterior lobe of the cerebellum and subcortical network (GM10), orbitofrontal–temporal network (GM11), and middle temporal network (GM12), and posterior cingulum (retrosplenial) bundle and angular WM network (WM1), inferior frontal WM network (WM2), corona radiata network (WM3), inferior parietal WM network (WM4), middle frontal WM network (WM5), anterior cingulum bundle network (WM6), occipital WM network (WM7), orbitofrontal WM network (WM8), middle cingulum bundle network (WM9), precentral/postcentral WM network (WM10), brainstem network (WM11), posterior thalamic radiation and posterior cingulum bundle network (WM12), cerebellum WM network (WM13), and inferior longitudinal fasciculus network (WM14)

| **Studies** | **Imaging**  **Modality** | **Analysis method** | **Correction for multiple comparisons** | **Content** | | | | | |
| --- | --- | --- | --- | --- | --- | --- | --- | --- | --- |
| Jin et al., 2022 | rs-fMRI | fALFF  whole-brain | voxel-based:  P < 0.001  FEW corrected VOXEL > 100 | **Significant differences in regional fALFF between the odontogenic pain caused by orthodontic separator and control groups** | | | | | |
|  |  |  |  | **Region BA side** | **MNI [mm]**  **x y z** | | | **Maximum *T* value** | **Cluster size (voxels)** |
|  |  |  |  | **increased fALFF in odontogenic group relative to control group** | | | | | |
|  |  |  |  | left cerebellum |  |  |  |  |  |
|  |  |  |  | bilateral inferior temporal gyrus |  |  |  |  |  |
|  |  |  |  | **decreased fALFF in odontogenic group relative to control group** | | | | | |
|  |  |  |  | medial prefrontal cortex |  |  |  |  |  |
|  |  |  |  | left anterior cingulate cortex |  |  |  |  |  |
|  |  |  |  | bilateral angular gyrus |  |  |  |  |  |
|  |  |  |  | left inferior parietal cortex |  |  |  |  |  |
|  |  |  |  | middle temporal gyrus |  |  |  |  |  |
|  |  |  |  | miscellaneous cerebral regions |  |  |  |  |  |

* MNI, cluster size: NA

| **Studies** | **Imaging**  **modality** | **Analysis method** | **Correction for multiple comparisons** | **Content** | | | |
| --- | --- | --- | --- | --- | --- | --- | --- |
| Zhang et al. 2022 | rs-fMRI | Global and nodal topological approach | Bonferroni  correction  p < 0.01 | **Regions with altered nodal centralities in subjects with experimental orthodontic pain compared with control subjects.** | | | |
|  |  |  |  | **Region side** | **Betweenness** | **Degree** | **Efficiency** |
|  |  |  |  | Superior frontal gyrus (lateral) R | 5.00* | 3.40 | 3.95 |
|  |  |  |  | Superior frontal gyrus (medial) R | 3.04 | 4.30 | 4.53* |
|  |  |  |  | Inferior frontal gyrus (ventral) R | 1.87 | 4.15 | 4.33* |
|  |  |  |  | Precentral gyrus (head and face region) R | 5.45* | 3.43 | 4.00 |
|  |  |  |  | Parahippocampal gyrus (entorhinalcortex)  R | 3.53 | 4.49 * | 4.13 |
|  |  |  |  | Postcentral gyrus R | 4.46* | 1.43 | 1.85 |
|  |  |  |  | Insular gyrus R | 5.15* | 3.90 | 4.52* |
|  |  |  |  | Caudal hippocampus R | 1.65 | 5.43 * | 4.62 |
|  |  |  |  | Occipital thalamus R | 2.98 | 6.91* | 6.57* |
|  |  |  |  | Ventral caudate L | 2.02 | 4.48* | 4.64 |
|  |  |  |  | Caudal temporal thalamus L | 3.10 | 5.76* | 5.22* |
|  |  |  |  | Parahippocampal gyrus (entorhinal cortex) L | 2.59 | 5.09* | 5.26* |
|  |  |  |  | Parahippocampal gyrus (posterior area) L | 3.48 | 5.28* | 4.82 |
|  |  |  |  | Middle cingulate gyrus R | -3.91 | -4.47* | -4.42* |
|  |  |  |  | Superior temporal gyrus (caudal) L | -4.50 * | -6.42* | -6.41* |
|  |  |  |  | Middle temporal gyrus (dorsolateral) L | -4.94 * | -7.53* | -7.34 |
|  |  |  |  | Inferior temporal gyrus (ventrolateral) L | -0.56 | -5.03* | -4.72* |
|  |  |  |  | Postcentral gyrus L | -1.33 | -4.58* | -4.19 |
|  |  |  |  | Middle cingulate gyrus L | -4.91* | -4.82* | -5.08* |
|  |  |  |  | Middle ventral occipital cortex L | -0.96 | -4.42* | -3.95 |
|  |  |  |  | Inferior occipital gyrus L | -1.57 | -4.47* | -4.22 |
|  |  |  |  | Lateral superior occipital gyrus L | -2.98 - | 4.33* | -4.08 |
|  |  |  |  | Caudal temporal thalamus R | 4.59* | 5.66* | 5.46* |

* Regions were considered abnormal in participants with orthodontic pain than controls (p < 0.01, Bonferroni correction for multiple comparisons)

Common Abbreviations: FC: Functional Connectivity; ROI: regions of interest; NA: not available; 3D T1: three-dimensional T1- weighted anatomical image; BA: Brodmann’s Area; MNI: Montreal Neurological Institute coordinates; FEW corrected: Family-Wise Error corrected; FDR: False Discovery Rate, P: Participants with orthodontic separator, C: Control group
